# Quantitative Trait Loci for Yield and Yield-Related Traits in Spring Barley Populations Derived from Crosses between European and Syrian Cultivars

**DOI:** 10.1371/journal.pone.0155938

**Published:** 2016-05-26

**Authors:** Krzysztof Mikołajczak, Piotr Ogrodowicz, Kornelia Gudyś, Karolina Krystkowiak, Aneta Sawikowska, Wojciech Frohmberg, Andrzej Górny, Andrzej Kędziora, Janusz Jankowiak, Damian Józefczyk, Grzegorz Karg, Joanna Andrusiak, Paweł Krajewski, Iwona Szarejko, Maria Surma, Tadeusz Adamski, Justyna Guzy-Wróbelska, Anetta Kuczyńska

**Affiliations:** 1 Institute of Plant Genetics, Polish Academy of Sciences, Strzeszyńska 34, 60–479 Poznań, Poland; 2 Department of Genetics, Faculty of Biology and Environmental Protection, University of Silesia, Jagiellońska 28, 40–032 Katowice, Poland; 3 Institute for Agricultural and Forest Environment, Polish Academy of Sciences, Bukowska 19, 60–809 Poznań, Poland; Huazhong university of Science and Technology, CHINA

## Abstract

In response to climatic changes, breeding programmes should be aimed at creating new cultivars with improved resistance to water scarcity. The objective of this study was to examine the yield potential of barley recombinant inbred lines (RILs) derived from three cross-combinations of European and Syrian spring cultivars, and to identify quantitative trait loci (QTLs) for yield-related traits in these populations. RILs were evaluated in field experiments over a period of three years (2011 to 2013) and genotyped with simple sequence repeat (SSR) and single nucleotide polymorphism (SNP) markers; a genetic map for each population was constructed and then one consensus map was developed. Biological interpretation of identified QTLs was achieved by reference to Ensembl Plants barley gene space. Twelve regions in the genomes of studied RILs were distinguished after QTL analysis. Most of the QTLs were identified on the 2H chromosome, which was the hotspot region in all three populations. Syrian parental cultivars contributed alleles decreasing traits' values at majority of QTLs for grain weight, grain number, spike length and time to heading, and numerous alleles increasing stem length. The phenomic and molecular approaches distinguished the lines with an acceptable grain yield potential combining desirable features or alleles from their parents, that is, early heading from the Syrian breeding line (Cam/B1/CI08887//CI05761) and short plant stature from the European semidwarf cultivar (Maresi).

## Introduction

Barley (*Hordeum vulgare* L.) is an important cereal crop. Although it is known to adapt to a wide range of environments, varieties cultivated in Europe were bred under favourable conditions, leading to the narrowing of genotypic variation in yield and morphological traits related to yield, as well as in adaptation to stresses, especially to water deficit [1]. In the past decades, in Central and West Europe the frequency of periods with reduced water availability increased; spring droughts in 2003, 2006, 2007, 2008, and 2011 resulted in severe economic impacts on the agricultural sector (http://ies.jrc.ec.europa.eu). Moreover, climatic change predictions indicate a considerably increased likelihood of drought in the Mediterranean, and Central and South-East Europe. In response to these climatic changes, breeding programmes should aim at creating new cultivars with improved resistance to water scarcity, particularly as regards spring cereals, which are most vulnerable to drought, and breeders need to seek new donors of genetic material for breeding cultivars that are more tolerant to water deficit. Barley genotypes originating from low-rainfall areas with genetically conditioned adaptability to drought appear promising for this purpose [2].

The selection of genotypes able to tolerate water deficit can be assisted by molecular markers. Progress in developing genetic maps of cereals, including barley, well-saturated with molecular markers, has allowed identification of the regions of the genome responsible for agronomically important traits. QTLs associated with yield and yield-related traits have been found on almost all barley chromosomes, but the number of QTLs, their additive effects, and their localisation on chromosomes have been dependent on populations and marker systems used for the construction of genetic maps [3–12]. Moreover, in most cases the QTL effects on agronomical traits were found to be affected by the QTL × environment interaction, while for selection QTLs with effects stable across environments are more useful [7–10,13–15].

Different marker systems have been applied for the construction of genetic maps [16–19]. More recently, simple sequence repeats (SSR) [19–22], single nucleotide polymorphism (SNP) [7,23–27] and diversity arrays technology (DArT) [19,20,28] markers have been widely applied in the construction of well-saturated genetic maps of barley. Availability of multiple mapping populations allowed the individual maps to be integrated into consensus maps with increased marker coverage [22,23,29,30]. Recently, an integrated physical, genetic, and functional sequence resource was reported by the International Barley Genome Sequencing Consortium [31]. These achievements provide opportunities for the development of innovative approaches for the use of genetic information hosted by public databases and, vice versa, the discovery of genome-wide SNP-QTL linkage helps in annotating barley genome sequence variants and exploring the mechanisms of gene regulation.

Barley populations derived from crosses between cultivars of diverse geographical origin have been frequently used to combine genetic factors involved in the expression of yield-forming traits [7,12,32]. We developed three recombinant inbred lines (RILs) barley populations from crosses between European and Syrian cultivars which provided the materials for the research project POLAPGEN-BD on resistance to drought in cereals, especially in barley. In the project, a systems approach to the problem of barley resistance to water shortage is applied. The objective of the present study was to detect the genetic factors determining yield potential in Syrian barley genotypes adapted to dry environments and to search for QTLs useful for selection of barley genotypes with desirable alleles for yield-related traits from both parents.

## Material and Methods

### Plant materials and experimental trials

#### Materials

Three populations of RILs derived from crosses between European and Syrian spring barley (*Hordeum vulgare* L.) cultivars—Maresi × Cam/B1/CI08887//, Lubuski × Cam/B1/CI08887//CI05761, and Georgie × Harmal (hereafter referred to as MCam, LCam and GH populations, respectively)—were used in our studies. Maresi is a German semidwarf cultivar with the pedigree Cebeco-6801/GB-1605//HA-46459-68, Lubuski is an old Polish cultivar derived from the Heines-Haisa/Skrzeszowicki hybrid, Georgie is a British cultivar with the pedigree Vada/Zephyr; Cam/B1/CI08887//CI05761 (hereafter referred to as CamB) and Harmal are a Syrian breeding line and cultivar, respectively, adapted to dry environments. Parental genotypes were chosen on the basis of earlier studies conducted by Górny and co-workers [1,33,34], in which tolerance to reduced water and nutrient supply and some physiological features were examined. The Syrian genotypes were supplied to Dr A. Górny (Institute of Plant Genetics PAS, Poznań) by Drs S. Grando and S. Ceccarelli from the Syrian ICARDA in Aleppo, and European cultivars from the collection of IPG PAS Poznań. RILs were derived by the single-seed descent technique [35] until F_8_ generation. About 150 lines were developed in each cross-combination, of which 100 were randomly chosen for the experiments.

#### Field experiments

Three hundred RIL lines and their parental cultivars were evaluated at IPG PAS. No specific permissions were required for this location (Cerekwica experimental station, Western Poland, 52°31′16″N, 16°41′30″E) and our field studies did not involve endangered or protected species. Populations MCam and LCam were investigated from 2011 to 2013, and population GH in 2012 and 2013. Sowing dates were: April 14 in 2011, April 10 in 2012 and April 8 in 2013. Experiments were established in a completely randomised design with three replications (plot size 1 m^2^, sowing rate 300 seeds per m^2^) on luvisol-type soil (according to the World Reference Base for Soil Resources 2006); each year fertiliser was added according to the soil-test recommendations for the cultivation of fodder barley.

The heading date (51 in BBCH scale [36]) was noted during vegetation, and at maturity plant height, length of main spikes, grain number per spike, grain weight per spike, 1000-grain weight, and grain yield per plot were observed. The observed traits and the methods of measurement are presented in Table 1.

**Table 1:** Agronomic traits observed in the experiments.

Trait(unit)	Abbreviation	Methods of measurement
Heading stage (days)	HS	Number of days from sowing to the beginning of heading—approximately 50% of spikes in a plot were in the growth stage 51 according to the BBCH scale
Length of main stem (cm)	LSt	Measured from soil surface to the tip of the spike (without awns)
Length of main spike (cm)	LSp	Measured from the base of spike to the tip of the terminal spikelet (without awns)
Number of grains per main spike	NGS	Counted on the basis of 20 randomly selected main spikes from each plot
Grain weight per main spike (g)	GWS	Average weight of hand-threshed grain from 20 randomly selected main spikes from each plot
1000-grain weight (g)	TGW	1000 × weight of one grain averaged for weight of grains from 20 main spikes
Grain yield (g)	GY	Weight of grain combine-harvested per plot

#### Agro-meteorological conditions

**Fig 1:**
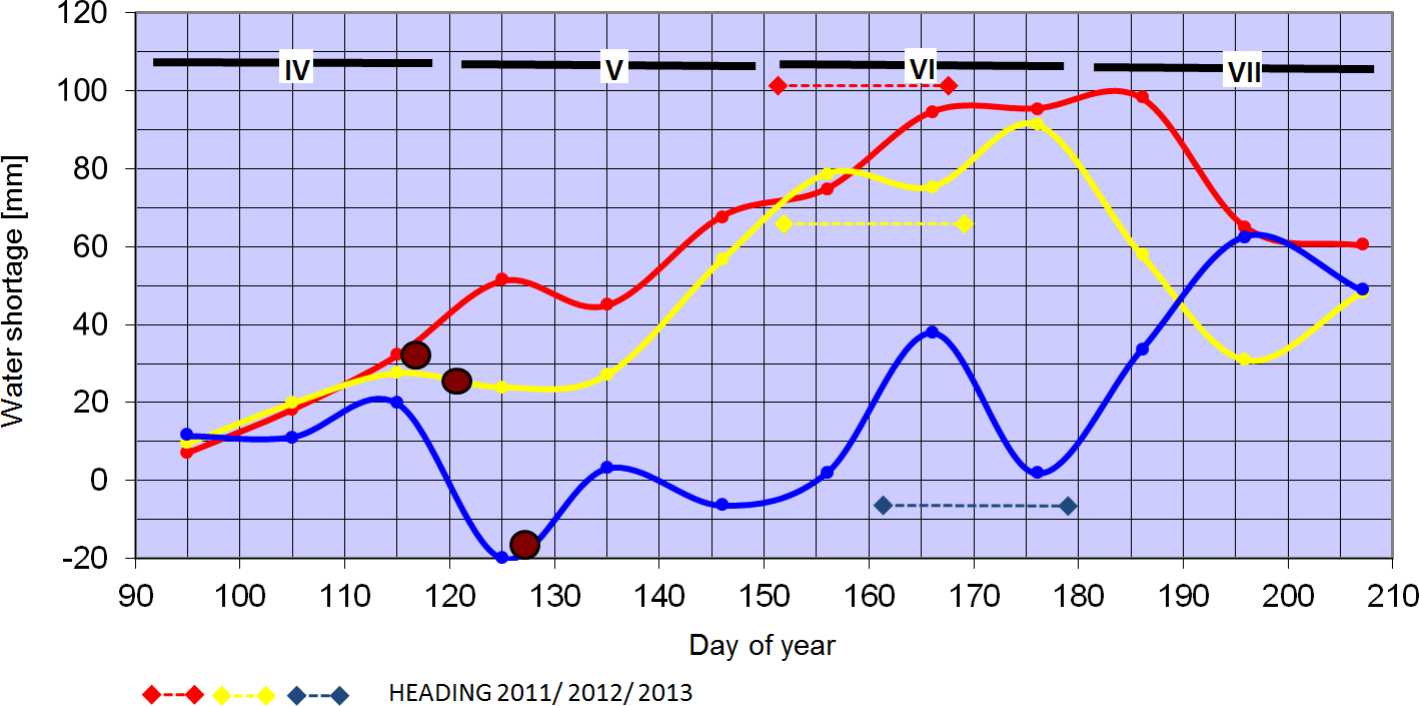
Profiles of cumulative water shortage (evapotranspiration—precipitation) at Cerekwica during period IV–VII in years 2011–2013: 2011- red; 2012—yellow; and 2013—blue; brown dot–germination.

From 2011 to 2013 agro-meteorogical parameters were measured at Cerekwica and four sites located around Cerekwica. Every year, air temperature, precipitation, and soil water potential were recorded from 1 April to 30 July. The mean values of air temperature, deficit of water vapour pressure, and precipitation in particular years differed slightly, but the courses of the parameters were different (S1 Table). April was dry in all three years, especially in 2011 when the total monthly rainfall (7.6 mm) was only 18% of the long-term average (43.1 mm), which resulted in increasing water scarcity (evapotranspiration minus precipitation; the real evapotranspiration was calculated on the basis of energy balance of ecosystem or landscape and agrometeorological index expressing impact of meteorological conditions and plant development stage [37]). In 2011 unfavourable conditions for plant growth occurred also in May when there was no precipitation in the first 10-day period; precipitation in the next two 10-day periods amounted to 31.2 mm and 6.8 mm. The permanent build-up of water shortages in 2011 was observed up to the first 10-day period of July, when their level reached as much as 100 mm (Fig 1). Better weather conditions prevailed in 2012, when total precipitation in May was only 11.1 mm less than the long-term average. The difference in hygro-thermal conditions between years is shown in Fig 1 as cumulative water shortage.

In 2013, the relatively low temperature in April caused a delay in sowing and delayed germination, resulting in late heading. However, abundant and fairly evenly distributed rainfall provided good plant development in May and June. In the first and second 10-day periods in July, extremely low precipitation (4.4 and 4.7 mm) was noted which was unfavourable for grain filling (the amount of rainfall in July was 65% of the long-term average) (S1 Table).

### Genotyping

Total genomic DNA of all RILs and parental cultivars was extracted from young leaf tissue using a C-TAB (cetyltriammonium bromide) method developed by Doyle and Doyle [38] with minor modifications. After quantification of DNA concentration using a NanoDrop ND-1000 Spectrophotometer (NanoDrop Technologies, Wilmington, DE, USA), the samples were diluted to 50 ngμl^−1^ in sterile water. Two types of DNA markers, SSR and SNP, were scored.

For SSR genotyping, a total of 245 SSR loci were screened for the polymorphism between the parents of the MCam, LCam, and GH mapping populations. The SSR markers were selected from the previously published SSR-based genetic maps of barley [18,22] and were evenly distributed on each of the seven barley chromosomes. SSR amplification reactions were carried out in a thermocycler (Biometra, Goettingen, Germany) in a 10 μl reaction volume containing 62.5 ng of genomic DNA, 1× PCR buffer, 0.2 mM dNTPs (Promega), 0.3 μM forward primer (Sigma-Aldrich), 0.3 μM IRD800 reverse primer (IBB PAS, Warsaw, Poland), and 0.1 U Taq polymerase (DyNAzymeTMII, Finnzymes, OY, Finland). PCR reaction conditions varied depending on the amplified SSR locus, and were used after Ramsay et al. [18] and Varshney et al. [22], or were slightly modified. Amplification products were visualised by electrophoresis in 6% denaturing polyacrylamide gels (acrylamide/bisacrylamide 19:1 solution, Sigma-Aldrich; 7 M urea, AppliChem; 1× TBE buffer) using Li-Cor 4300 DNA Analyzer (Li-Cor, Lincoln, NE; 1300 V, 30 mA, 30 W, and at a medium speed for laser scanning). SSR markers that revealed the polymorphism between the parental cultivars were used for the genotyping of the MCam and GH mapping populations.

For SNP genotyping, frozen DNA samples were submitted to the Southern California Genotyping Consortium (SCGC), Illumina BeadLab at the University of California, Los Angeles (UCLA), and genotyped with the 1536-SNP barley oligonucleotide pool assay (BOPA1) [23] using the Illumina Golden Gate Bead Array SNP detection platform. Genotyping was conducted for all parental cultivars, 94 RILs for MCam and LCam, and 100 RILs for GH (six lines from MCam and LCam population were removed because of technical limitations). Genotype calls were inspected, and all SNPs that were nonpolymorphic between the parents or produced ambiguous results were removed from the analysis.

### Individual and consensus map construction

Individual maps of all three mapping populations as well as a consensus map were constructed with JoinMap 3.0 [39]. For each marker, the deviation from the expected 1:1 segregation ratio was tested with the chi-square test at significance level *α* = 0.05. Marker linkage groups for each of the three mapping populations were selected at logarithm of odds (LOD) scores ranging from 6 to 11. Marker order analysis was conducted with a recombination frequency (REC) threshold value ≤ 0.4. The obtained marker order within each linkage group was compared with the marker order from the reference SSR and SNP barley maps [22,23] and the discrepancies between compared marker orders were analysed. The markers which were mapped to incorrect regions of the chromosomes were removed from the mapping and the marker order was calculated again. This procedure was repeated until all obvious discrepancies were eliminated. Estimated genetic distances between markers were based on the recombination fraction using the Kosambi mapping function [40]. For the construction of a consensus map, linkage groups from the individual maps which represented the particular chromosome and had at least two loci in common (so-called anchor markers) were integrated by applying the ‘combine groups for map integration’ function of JoinMap. The locus order and genetic distances within each integrated linkage group were calculated with the same parameters as for the construction of the linkage groups on the individual maps. Then, the integrated linkage groups were compared again with the reference barley maps with regard to the order of markers, and the discrepancies were analysed and eliminated.

### Data analysis

Observations of phenotypic traits were subjected to analysis of variance in the mixed model with fixed effects of year and random effects of line and interaction of lines with years. A residual maximum likelihood (REML) algorithm was used to estimate variance components for random effects and the *F*-statistic was computed to assess the significance of fixed effects. Broad sense heritability was computed from appropriate variance components. Mean values computed independently for RILs in all years were used for the construction of principal component bi-plots. All these analyses were performed with Genstat 16 [41]. Stability assessment for RILs was performed using the analysis of genotype × environment interaction in the mixed linear model as described by Caliński et al. [42] and implemented in the computer program Sergen [43].

QTL localisation in the consensus linkage map was performed for each population and trait independently with the method described by Malosetti et al. [44] implemented in Genstat 16 [41]. Only lines with less than 20% of missing genotype data were included (94, 88, and 99 lines for populations MCam, LCam, and GH, respectively). After selection of the best covariance structure for modelling genetic correlations among environments, interval mapping was performed with the step size of 2 cM by first selecting candidate QTLs and then using them iteratively as co-factors until the list remained unchanged. The threshold for the −log_10_(*P*-value) statistic was computed with the method of Li and Ji [45] to ensure that the genome-wide error rate was smaller than 0.01. The windows for not selecting two close QTLs and for exclusion of co-factors were set at 10 and 30 cM, respectively. Selection of the set of QTL effects in the final model was done at *P* < 0.05; the *P*-values for the Wald statistic were computed as the mean from the values obtained by adding and dropping the QTL main and interaction effects in the model. QTLs were defined as belonging to one map region if the ±1.8 cM intervals around their positions overlapped (1.8 cM being half of the average length of two LOD support interval computed for all QTLs; see Xu [46]).

QTL annotation was achieved by mapping all SNP sequences taken from Close et al. [23] (supplementary material file BOPA1 SNP 1471-2164-10-582-S19.xls) to barley genome space in Ensembl Plants (ver. 082214v1, reference repeat masked sequence Hordeum_vulgare.082214v1.28.dna_rm.toplevel.fa, NCBI Blast for Windows with maximum EValue = 1e-060, minimum 95% identity of the SNP sequence). The SNP mapping positions were used to obtain a projection of two LOD QTL support intervals onto the genomic sequence; all genes located in projected intervals were listed and annotated using Gene Ontology terms.

## Results

### Phenotypic evaluation of RIL populations

**Fig 2:**
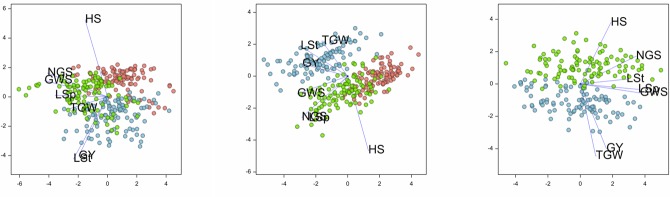
Principal component biplots constructed for phenotypic traits observed in MCam, LCam and GH populations in years 2011 (red), 2012 (blue), and 2013 (green).

Differences in agro-meteorological conditions between years considerably influenced grain yield and other analysed traits. The bi-plots in Fig 2 summarise how the traits contributed in a correlated way to differences between features of lines in different years. For MCam and LCam, the plots were similar and characterised by later heading (HS) in all years. Agro-meteorological conditions in 2012 were specifically good for grain yield and length of stem (GY, LSt), whereas in 2013 they were propitious for the development of spike traits: length, number of grains, and grain weight (GWS, LSp, NGS). For the GH population, 2013 differed from 2012 by later heading (HS), as in the case of LCam and MCam, and was less favourable for 1000-grain weight and grain yield (TGW, GY).

European and Syrian parents of RIL populations differed significantly in all the studied traits. Early heading and a low yield potential are the main features that distinguished the Syrian genotypes from the European ones. The heading time of CamB and Harmal was on average nine days earlier than that for Georgie, Lubuski, and Maresi (S2 Table). Grain yield of Syrian lines constituted, on average over the years, something above 60% of that for European cultivars. However, in 2011, when a strong water deficit occurred in spring, the grain yield of CamB was relatively higher than that in 2012 and 2013 and amounted to 76.7% of mean yield for Lubuski and Maresi. Harmal and CamB were characterised by shorter spikes and lower number and weight of grains per spike than European cultivars (S2 Table).

The lines of all RIL populations were significantly differentiated in earliness, plant height, and yield and its structure. Differences between the earliest and latest heading lines in three years amounted to 9 days in GH, 10–12 days in LCam, and 11–15 days in MCam populations. Grain yield was strongly influenced by environments—in 2011 it was generally about four times lower than that in 2012 and 2013. In each year, the best lines yielded three to five times as much as the worst ones, but differentiation of lines in yielding was most apparent in 2011. Grain yield was the trait for which coefficient of variation was the highest and ranged from 15.70% for MCam in 2013 to 23.45% for LCam in 2013. Analysis across years also revealed a strong differentiation of RIL populations concerning spike traits: length, number of grains, and grain weight per spike, for which the coefficients of variation were between 7.67% and 18.11% (S1 Table).

Analysis of variance indicated significant effects of year for all populations and traits (*P* < 0.001) with the exception of TGW in MCam, and GWS and LSp in the GH population. Variance components for lines were, in general, similar in all three populations, but in the GH population greater variance was noted for grain yield and relatively smaller variance for days to heading, and in LCam smaller variance was noted for length of main stem and number of grains per spike. The variance component for lines × years interaction was smaller than that for lines in almost all cases, with the exception of LSt in LCam and GY in all three populations; for the latter trait, the interaction component was several times higher than that for lines (Table 2).

**Table 2:** ANOVA results, variance components, and broad sense heritability estimates for phenotypic traits observed in RIL populations.

Trait name(Symbol)	Population	Significance of differences between years (*P*-value for *F*-test)	Variance component for lines (s.e.)	Variance component for interaction (lines × years) (s.e.)	Broad sense heritability in %
Heading stage(HS)	MCam	<0.001	8.90 (1.39)	2.14 (0.26)	91.23
	LCam	<0.001	6.55 (1.03)	1.58 (0.20)	90.76
	GH	<0.001	1.54 (0.35)	1.33 (0.23)	66.02
Length of main stem(LSt)	MCam	<0.001	38.79 (6.22)	6.67 (1.52)	88.94
	LCam	<0.001	4.63 (1.49)	6.64 (1.66)	47.02
	GH	<0.001	22.70 (4.48)	4.56 (2.36)	74.33
Length of main spike(LSp)	MCam	<0.001	0.495 (0.078)	0.037 (0.017)	90.50
	LCam	<0.001	0.225 (0.041)	0.054 (0.019)	79.46
	GH	0.888	0.713 (0.112)	0.035 (0.023)	98.16
Number of grains per main spike(NGS)	MCam	<0.001	4.85 (0.75)	0.47 (0.14)	91.70
	LCam	<0.001	2.02 (0.42)	1.61 (0.27)	69.49
	GH	<0.001	6.04 (0.93)	0.14 (0.14)	93.13
Grain weight per main spike(GWS)	MCam	<0.001	0.0184 (0.0029)	0.0029 (0.0007)	89.89
	LCam	<0.001	0.0092 (0.0017)	0.0037 (0.0008)	77.97
	GH	0.045	0.0201 (0.0031)	0.0013 (0.0007)	90.04
1000-grain weight(TGW)	MCam	0.098	5.03 (0.95)	2.91 (0.49)	75.89
	LCam	<0.001	5.36 (0.99)	2.17 (0.49)	77.65
	GH	<0.001	8.44 (1.65)	3.08 (0.82)	74.96
Grain weight per m^2^(GY)	MCam	<0.001	1079 (566)	5975 (757)	30.19
	LCam	<0.001	1684 (752)	8212 (945)	35.02
	GH	<0.001	2986 (1147	6244 (1148)	42.66

Broad sense heritability coefficients for the studied traits were generally similar in all the populations. Among the traits, the lowest heritability, below 50%, was found for grain yield in GH, LCam, and MCam (30.19–42.66%), and for length of main stem in LCam (47.02%). Heritability coefficients for 1000-grain weight in all three populations were very similar and amounted to about 75%, whereas for the rest of the observed characteristics they ranged from 47.02% (LSt in LCam) to 93.13% (NGS in GH). Comparison of half-sibling populations LCam and MCam showed slight differences in the coefficients of heritability for grain number and grain weight per spike, which were lower in LCam (Table 2).

### Construction of genetic map

Among the 245 screened SSR markers of good quality, in MCam and GH populations exactly the same number of markers (115, 46.9%) showed polymorphism between parents. Of the 1536 SNPs represented on BOPA1, markers 1441, 1392, and 1536 were of good quality in MCam, GH, and LCam populations, respectively. The corresponding fraction of polymorphic SNPs was slightly lower than that of SSRs, and was found to be 533 (36.9%), 523 (37.5%), and 462 (30.1%). Segregation distortions were observed in all three mapping populations, and they were the most pronounced in the LCam population. There was a significant deviation from the expected allele segregation ratio in a total of 42.9% of markers in MCam, 36.6% in GH, and 61.7% in LCam. Most of the markers that exhibited the distorted segregations were skewed toward the European parental cultivar (76.2% in MCam, 79.8% in GH, and 54.6% in LCam) and they were mostly gathered in clusters on the chromosomes. The largest clusters of the skewed markers were found on 1H in MCam, on 2H in GH, and on 3H.1, 4H, and 5H.2 in LCam.

Individual genetic maps were created for each of the three RIL populations foregoing the consensus map construction. Table 3 shows detailed information regarding the number of mapped markers and average inter-loci distance for all three individual linkage maps and the consensus map. The final consensus linkage map contained 819 unique loci including 387 anchor markers which were common to at least two mapping populations (S1 Fig). Altogether, 13 linkage groups were defined, as the chromosomes 1H and 3H were split into two linkage groups, and chromosomes 5H and 7H into three linkage groups. The markers were uniformly distributed in all linkage groups, except for a few gaps (~ 10.0 cM) that existed on the chromosomes 1H, 2H, 6H, and 7H.). The obtained marker orders were in good agreement with other published barley integrated maps, and slight differences in marker order were observed mainly in the centromeric regions of the chromosomes.

**Table 3:** Characteristics of the three individual linkage maps and the consensus barley map.

	Map			
	MCam	GH	LCam	Consensus
No. of mapped markers	548	562	485	819
No. of mapped SSR markers	100	98	78	117
No. of mapped SNP markers	448	464	485	702
Map length [cM]	873.9	787.8	686.0	953.8
Average interloci distance [cM]	1.6	1.4	1.4	1.2

### QTL analyses

In total, 89 QTLs were detected for all seven traits in three RIL populations; 53 of them were found in 12 regions of the genome (named A to L). The numbers of QTLs in populations MCam, LCam, and GH were 36, 21, and 32, respectively. The largest number of QTLs was found for TGW (21) and the smallest for GY (5). The percentage of QTLs with significant QTL × environment interaction for MCam, LCam, and GH was 36.0%, 52.2%, and 12.5%, respectively. The largest percentage of QTLs with QTL × environment interaction was found for GY (60%) and HS (53%), the smallest for NGS (8%). The QTLs for individual traits were uniformly distributed among populations, with the exception of LSp, where most of the QTLs were detected in MCam (8) and the fewest in LCam (1).

Out of 15 QTLs detected for days to heading six were found on chromosome 2H (Table 4). The main effects in each population showed the QTLs located in region B: in LCam and GH at the same SNP 5880–2547 (QHS.LC-2H-1, QHS.GH-2H-1), in MCam at SSR marker GBM1214 (QHS.MC-2H). These QTLs explained a large proportion of phenotypic variation in each population. QTLs found in LCam and MCam showed interaction with environments. On the 2H chromosome, two other QTLs (QHS.GH-2H-2 and QHS.GH-2H-3) were detected in the GH population, though with small effects, both located in region F and close to each other at a distance of less than 2 cM. All QTLs on 2H (except for one in LCam) possessed Syrian parental genotypes alleles causing the decrease of the number of days to heading. A significant QTL (QHS.MC-3H.1-2) with a stable effect of CamB allele decreasing time to heading was also found in the linkage group 3H.1 (region H), but only in the MCam population, whereas the decreasing effect of the Harmal allele was detected in 7H.2 (region L—QHS.GH-7H.2). The CamB allele also contributed to increasing time to heading; QTLs with significantly positive effects were detected in the linkage group 5H.3 (region K) in both LCam and MCam populations (QHS.MC-5H.3 and QHS.LC-5H.3) at the same SNP 314–559. Both QTLs showed interaction with the environment and their additive effects were the highest in 2012 and similar in both populations. In that year, these QTLs explained 14.57% and 21.66% of phenotypic variation in MCam and LCam, respectively.

**Table 4:** QTLs identified in RIL populations MCam, LCam, and GH for observed phenotypic traits. QTL strength measured by the statistic −Log_10_(*P*-value) with *P*-values for Wald test computed based on accumulated analysis of variance.

Population	Trait	Linkage group	QTL ID	Position [cM]	Region ^(d)^	Marker	Shift from marker to QTL position [cM]	−Log_10_(*P*-value)	QTL × E interaction	Additive effect [trait units] ^(a)^			Percent of variance explained by QTL in years [%] ^(b)^			Percent of variance explained by QTL main effect and interaction [%] ^(c)^		
										2011	2012	2013	2011	2012	2013	Main effect	Interaction	Total
MCam	Number of grains per main spike	1H.1	QNGS.MC-1H.1	47.95		11603–445	0	7.32	No	0.96	0.96	0.96	15.77	13.47	14.74	8.97	0.00	8.97
MCam	1000-grain weight	1H.1	QTGW.MC-1H.1	53.41	A	6655–978	0	2.56	Yes	n.s.	−0.67	n.s.	0.17	3.53	1.90	0.00	1.55	1.55
MCam	Grain weight per main spike	1H.1	QGWS.MC-1H.1	55.27	A	2036–1027	−1.86	4.55	Yes	n.s.	−0.04	0.03	0.19	5.80	4.09	0.00	1.61	1.61
MCam	Length of main spike	1H.2	QLSp.MC-1H.2	11.48		Bmac0154	9.82	3.16	Yes	n.s.	n.s.	0.21	0.49	0.18	5.14	0.00	1.05	1.05
GH	1000-grain weight	1H.2	QTGW.GH-1H.2-1	16.05		1347–1061	1.97	5.84	Yes	-	−1.94	n.s.	-	25.34	0.10	2.21	3.29	5.50
GH	1000-grain weight	1H.2	QTGW.GH-1H.2-2	27.76		3786–2204	0	3.04	Yes	-	0.69	−0.85	-	3.21	5.59	0.00	2.14	2.14
GH	Length of main stem	1H.2	QLSt.GH-1H.2	34.41		557–1297	0	5.62	No	-	1.52	1.52	-	6.57	5.51	5.85	0.00	5.85
GH	Number of grains per main spike	1H.2	QNGS.GH-1H.2	44.57		Bmag0579	0	8.84	No	-	0.95	0.95	-	12.97	12.37	12.56	0.00	12.56
MCam	Grain weight per main spike	2H	QGWS.MC-2H-1	9.15	B	5880–2547	−1.59	30.60	No	−0.12	−0.12	−0.12	54.30	57.50	50.70	31.51	0.00	31.51
LCam	1000-grain weight	2H	QTGW.LC-2H-1	10.74	B	5880–2547	0	3.05	Yes	−1.03	n.s.	n.s.	10.58	1.45	0.16	0.00	1.67	1.67
LCam	Grain weight per m^2^	2H	QGY.LC-2H	10.74	B	5880–2547	0	8.91	Yes	n.s.	−91.06	n.s.	2.56	43.16	4.97	0.35	17.77	18.12
LCam	Grain weight per main spike	2H	QGWS.LC-2H	10.74	B	5880–2547	0	5.56	No	−0.06	-0.06	−0.06	27.58	16.25	24.63	14.02	0.00	14.02
LCam	Heading stage	2H	QHS.LC-2H-1	10.74	B	5880–2547	0	31.86	Yes	−2.93	−1.22	−3.12	82.80	24.62	103.85	37.08	5.40	42.48
GH	Heading stage	2H	QHS.GH-2H-1	10.74	B	5880–2547	0	12.37	No	-	−1.68	−1.68	-	104.34	78.24	21.25	0.00	21.25
MCam	Length of main spike	2H	QLSp.MC-2H-1	10.74	B	5880–2547	0	8.25	Yes	−0.19	−0.44	−0.51	7.57	29.42	31.72	11.90	2.43	14.33
MCam	Length of main stem	2H	QLSt.MC-2H	10.74	B	5880–2547	0	5.39	Yes	4.44	n.s.	3.83	81.15	4.65	23.06	0.08	2.09	2.17
MCam	Number of grains per main spike	2H	QNGS.MC-2H	10.74	B	5880–2547	0	37.62	No	−2.22	−2.22	−2.22	83.98	71.73	78.49	51.11	0.00	51.11
LCam	Number of grains per main spike	2H	QNGS.LC-2H-1	10.74	B	5880–2547	0	3.55	No	−0.95	−0.95	−0.95	27.64	15.42	18.99	12.56	0.00	12.56
MCam	1000-grain weight	2H	QTGW.MC-2H-1	11.59	B	GBM1214	0	11.73	Yes	−2.27	n.s.	−0.99	52.78	0.00	12.32	6.62	4.97	11.60
MCam	Heading stage	2H	QHS.MC-2H	11.59	B	GBM1214	0	71.49	Yes	−3.14	−1.40	−3.82	88.76	21.61	91.32	38.77	5.35	44.12
MCam	Grain weight per m^2^	2H	QGY.MC-2H	16.01		Bmag0692	0	5.86	Yes	n.s.	−64.67	n.s.	0.00	25.84	3.55	0.90	13.70	14.60
GH	Grain weight per main spike	2H	QGWS.GH-2H-1	27.84	C	2417–924	−13.09	6.45	No	-	−0.08	−0.08	-	22.28	24.90	6.55	0.00	6.55
GH	Length of main spike	2H	QLSp.GH-2H-1	27.84	C	2417–924	−13.09	5.18	No	-	−0.39	−0.39	-	21.10	15.06	5.57	0.00	5.57
GH	Number of grains per main spike	2H	QNGS.GH-2H-1	35.32	D	2417–924	−5.61	3.06	No	-	−0.70	−0.70	-	7.04	6.71	3.23	0.00	3.23
MCam	Length of main spike	2H	QLSp.MC-2H-2	37.00	D	2417–924	−3.93	4.42	No	0.23	0.23	0.23	11.07	8.00	6.28	4.63	0.00	4.63
MCam	Grain weight per main spike	2H	QGWS.MC-2H-2	48.35	E	2580–1456	0	11.36	No	−0.05	−0.05	−0.05	8.80	9.32	8.22	7.71	0.00	7.71
MCam	1000-grain weight	2H	QTGW.MC-2H-2	49.79	E	2580–1456	1.44	4.11	No	−0.78	−0.78	−0.78	6.18	4.76	7.64	3.91	0.00	3.91
GH	Grain weight per m^2^	2H	QGY.GH-2H	49.79	E	2580–1456	1.44	7.34	No	-	−48.32	−48.32	-	16.12	30.57	16.55	0.00	16.55
GH	Length of main stem	2H	QLSt.GH-2H	49.79	E	2580–1456	0	8.34	No	-	−1.99	−1.99	-	11.29	9.47	8.57	0.00	8.57
GH	Grain weight per main spike	2H	QGWS.GH-2H-2	55.72	F	Bmag0720	0	15.61	No	-	−0.10	−0.10	-	37.50	41.90	30.86	0.00	30.86
GH	Heading stage	2H	QHS.GH-2H-2	55.72	F	Bmag0720	0	5.72	Yes	-	n.s.	−0.77	-	0.94	16.31	2.62	2.69	5.31
GH	Length of main spike	2H	QLSp.GH-2H-2	55.72	F	Bmag0720	0	16.93	No	-	−0.62	−0.62	-	53.15	37.92	36.30	0.00	36.30
GH	Heading stage	2H	QHS.GH-2H-3	57.48	F	7489–442	1.32	2.78	No	-	−0.44	−0.44	-	7.31	5.48	1.82	0.00	1.82
GH	Number of grains per main spike	2H	QNGS.GH-2H-2	57.48	F	7489–442	1.32	11.59	No	-	−1.74	−1.74	-	43.98	41.97	27.68	0.00	27.68
LCam	Heading stage	2H	QHS.LC-2H-2	80.13		ConsensusGBS0705-1	0	2.74	No	0.40	0.40	0.40	1.56	2.69	1.73	1.00	0.00	1.00
MCam	Length of main spike	2H	QLSp.MC-2H-3	106.85		14832–296	−1.78	6.84	No	−0.26	−0.26	−0.26	14.49	10.48	8.23	7.15	0.00	7.15
GH	1000-grain weight	2H	QTGW.GH-2H	117.45		2822–739	0	8.16	No	-	−1.03	−1.03	-	7.09	8.24	6.74	0.00	6.74
MCam	Length of main spike	2H	QLSp.MC-2H-4	125.68		GBM1462	−1.46	2.81	Yes	n.s.	n.s.	−0.20	0.24	0.23	4.82	0.12	0.78	0.90
LCam	Number of grains per main spike	2H	QNGS.LC-2H-2	140.95		1344–930	−3.95	2.37	No	−0.69	−0.69	−0.69	14.47	8.07	9.94	3.39	0.00	3.39
LCam	1000-grain weight	2H	QTGW.LC-2H-2	144.90		1344–930	0	5.08	No	0.96	0.96	0.96	9.15	9.70	8.48	5.94	0.00	5.94
GH	1000-grain weight	3H.1	QTGW.GH-3H.1	0.00		GBM1280	0	5.27	No	-	−0.94	−0.94	-	5.90	6.85	3.97	0.00	3.97
MCam	Number of grains per main spike	3H.1	QNGS.MC-3H.1	8.94		3646–1984	0	5.53	No	0.55	0.55	0.55	5.24	4.47	4.90	3.47	0.00	3.47
MCam	Length of main spike	3H.1	QLSp.MC-3H.1	20.71		3886–313	0	5.93	No	0.23	0.23	0.23	11.17	8.08	6.34	7.29	0.00	7.29
MCam	Heading stage	3H.1	QHS.MC-3H.1-1	26.53	G	3688–1291	1.69	5.14	Yes	n.s.	n.s.	0.68	0.07	0.12	2.86	0.00	0.64	0.64
LCam	Number of grains per main spike	3H.1	QNGS.LC-3H.1	28.16	G	4593–2007	−1.74	11.92	Yes	n.s.	1.31	−1.09	0.00	29.32	25.22	0.00	10.99	10.99
LCam	Grain weight per main spike	3H.1	QGWS.LC-3H.1	29.90	G	4593–2007	0	8.19	Yes	n.s.	0.04	−0.05	0.29	7.25	14.45	0.00	6.21	6.21
GH	Length of main stem	3H.1	QLSt.GH-3H.1-1	47.26		5657–1187	0	12.33	No	-	3.25	3.25	-	29.93	25.11	26.24	0.00	26.24
LCam	Grain weight per m^2^	3H.1	QGY.LC-3H.1	67.12		4150–398	1.68	4.78	Yes	n.s.	−43.60	−60.43	0.21	9.89	24.05	0.00	10.52	10.52
GH	Length of main stem	3H.1	QLSt.GH-3H.1-2	79.49		GBM1090	0	6.89	No	-	-1.80	−1.80	-	9.16	7.69	6.23	0.00	6.23
MCam	1000-grain weight	3H.1	QTGW.MC-3H.1	102.19	H	ABC07496-pHv1343-02	−1.68	5.13	Yes	0.73	1.75	n.s.	5.45	24.18	1.39	4.05	1.93	5.98
MCam	Heading stage	3H.1	QHS.MC-3H.1-2	102.19	H	ABC07496-pHv1343-02	−1.68	12.30	No	−1.19	−1.19	−1.19	12.67	15.45	8.82	6.98	0.00	6.98
MCam	Length of main stem	3H.1	QLSt.MC-3H.1	105.75	H	5260–462	0	2.91	No	4.85	4.85	4.85	96.66	34.36	37.02	1.97	0.00	1.97
LCam	Length of main stem	3H.1	QLSt.LC-3H.1	106.62	H	4026–655	−1.2	3.90	No	1.65	1.65	1.65	37.47	9.41	10.68	0.60	0.00	0.60
GH	Grain weight per main spike	3H.2	QGWS.GH-3H.2	3.64		5008–2402	0	4.72	No	-	−0.04	−0.04	-	5.49	6.14	4.44	0.00	4.44
MCam	Grain weight per main spike	4H	QGWS.MC-4H	7.43	I	2055–947	−1.6	11.23	No	0.06	0.06	0.06	13.93	14.75	13.01	11.58	0.00	11.58
MCam	Number of grains per main spike	4H	QNGS.MC-4H	9.03	I	2055–947	0	10.42	No	0.83	0.83	0.83	11.69	9.98	10.92	7.73	0.00	7.73
LCam	Length of main stem	4H	QLSt.LC-4H	23.32		2769–1245	0	4.60	Yes	0.56	2.47	n.s.	4.29	21.07	0.94	6.07	3.68	9.75
GH	1000-grain weight	4H	QTGW.GH-4H	47.58		Bmac0181	9.29	6.77	No	-	1.26	1.26	-	10.71	12.45	5.41	0.00	5.41
LCam	1000-grain weight	4H	QTGW.LC-4H	52.16		ABC09432-1-1-160	0	6.40	No	−1.16	−1.16	−1.16	13.54	14.35	12.55	8.04	0.00	8.04
GH	Heading stage	4H	QHS.GH-4H	91.50		WMS6	−1.64	8.66	No	-	0.58	0.58	-	12.46	9.34	7.95	0.00	7.95
LCam	Heading stage	4H	QHS.LC-4H	101.11		954–1377	0	3.17	Yes	−0.48	n.s.	−0.76	2.25	0.04	6.19	1.27	0.75	2.01
MCam	Length of main stem	5H.1	QLSt.MC-5H.1	8.06	J	10207–1024	0	4.03	No	1.41	1.41	1.41	8.19	2.91	3.14	3.58	0.00	3.58
MCam	Grain weight per main spike	5H.1	QGWS.MC-5H.1	9.85	J	10207–1024	1.79	7.39	No	0.04	0.04	0.04	7.84	8.30	7.32	4.91	0.00	4.91
GH	1000-grain weight	5H.1	QTGW.GH-5H.1	12.74	J	847–1396	0	2.59	No	-	−0.56	−0.56	-	2.09	2.43	1.51	0.00	1.51
GH	Length of main stem	5H.2	QLSt.GH-5H.2	18.28		8320–955	0	6.59	Yes	-	n.s.	2.39	-	1.09	13.64	4.04	1.73	5.77
MCam	1000-grain weight	5H.3	QTGW.MC-5H.3-1	48.27		10047–338	0	3.85	No	−0.74	−0.74	−0.74	5.59	4.30	6.90	4.57	0.00	4.57
MCam	Length of main stem	5H.3	QLSt.MC-5H.3	56.98	K	4795–782	0	4.99	No	−1.74	−1.74	−1.74	12.44	4.42	4.76	4.88	0.00	4.88
MCam	Length of main spike	5H.3	QLSp.MC-5H.3	58.01	K	314–559	−1.02	10.14	No	−0.33	−0.33	−0.33	23.23	16.79	13.19	9.66	0.00	9.66
MCam	Heading stage	5H.3	QHS.MC-5H.3	59.03	K	314–559	0	7.53	Yes	n.s.	1.15	0.64	0.15	14.57	2.60	1.65	1.70	3.36
LCam	Heading stage	5H.3	QHS.LC-5H.3	59.03	K	314–559	0	9.41	Yes	0.72	1.14	n.s.	4.92	21.66	1.09	4.03	1.01	5.05
LCam	Length of main spike	5H.3	QLSp.LC-5H.3	61.54	K	ConsensusGBS0138-2	0	5.54	No	−0.22	−0.22	−0.22	18.36	12.46	9.40	8.64	0.00	8.64
LCam	1000-grain weight	5H.3	QTGW.LC-5H.3	71.50		ConsensusGBS0086-5	0	2.51	Yes	n.s.	−1.06	n.s.	2.44	12.00	0.00	1.47	1.36	2.83
MCam	1000-grain weight	5H.3	QTGW.MC-5H.3-2	76.50		1204–1104	0	4.62	No	0.75	0.75	0.75	5.76	4.43	7.12	3.99	0.00	3.99
GH	Heading stage	6H	QHS.GH-6H	42.18		ABC04725-1-1-254	0	3.42	No	-	0.36	0.36	-	4.71	3.53	3.42	0.00	3.42
GH	1000-grain weight	6H	QTGW.GH-6H	54.22		HVM31	0	12.08	No	-	−1.53	−1.53	-	15.68	18.22	10.56	0.00	10.56
LCam	1000-grain weight	6H	QTGW.LC-6H	77.41		ConsensusGBS0708-6	−1.65	7.72	No	1.24	1.24	1.24	15.27	16.19	14.16	9.95	0.00	9.95
MCam	Number of grains per main spike	6H	QNGS.MC-6H	84.28		1597–158	0	3.75	No	−0.48	−0.48	−0.48	3.85	3.29	3.60	2.07	0.00	2.07
LCam	1000-grain weight	7H.2	QTGW.LC-7H.2	10.33	L	5777–354	0	4.11	No	0.92	0.92	0.92	8.42	8.93	7.81	4.93	0.00	4.93
GH	Grain weight per m^2^	7H.2	QGY.GH-7H.2	11.99	L	ABC03024-1-3-368	0	7.33	No	-	−35.74	−35.74	-	8.82	16.72	9.62	0.00	9.62
GH	Length of main spike	7H.2	QLSp.GH-7H.2	11.99	L	ABC03024-1-3-368	0	7.44	No	-	−0.27	−0.27	-	10.27	7.33	9.03	0.00	9.03
GH	Grain weight per main spike	7H.2	QGWS.GH-7H.2	13.48	L	6353–524	−1.48	5.81	No	-	−0.04	−0.04	-	7.46	8.34	6.14	0.00	6.14
GH	Heading stage	7H.2	QHS.GH-7H.2	13.48	L	6353–524	−1.48	12.43	No	-	−0.66	−0.66	-	16.07	12.05	12.23	0.00	12.23
GH	Length of main stem	7H.2	QLSt.GH-7H.2	14.96	L	6353–524	0	4.11	No	-	−1.30	−1.30	-	4.78	4.01	4.10	0.00	4.10
GH	1000-grain weight	7H.2	QTGW.GH-7H.2	30.14		4679–1594	0	4.99	No	-	−0.86	−0.86	-	4.96	5.76	3.99	0.00	3.99
MCam	Number of grains per main spike	7H.2	QNGS.MC-7H.2	34.31		2669–1012	0	3.12	No	−0.40	−0.40	−0.40	2.70	2.30	2.52	1.84	0.00	1.84
MCam	Heading stage	7H.3	QHS.MC-7H.3	9.35		5796–1189	−1.85	3.29	Yes	0.44	n.s.	n.s.	1.78	1.76	0.27	0.09	0.35	0.44
MCam	1000-grain weight	7H.3	QTGW.MC-7H.3	29.93		EBmac0755	0	3.17	No	0.65	0.65	0.65	4.38	3.37	5.41	3.27	0.00	3.27
LCam	Grain weight per main spike	7H.3	QGWS.LC-7H.3	39.99		Bmac0156	0	2.91	Yes	n.s.	n.s.	0.03	0.13	0.88	6.75	0.00	1.95	1.95
MCam	Length of main spike	7H.3	QLSp.MC-7H.3	43.76		8923–707	0	11.57	No	0.30	0.30	0.30	19.24	13.91	10.93	17.57	0.00	17.57

(a) Additive effects: negative—alleles increasing trait value from Maresi. Lubuski. or Georgie; positive—alleles increasing trait value from Cam/B1/CI or Harmal

(b) Percentage of variance computed as the ratio of variance for effect and variance in given year; values larger than 100% indicate overestimation of additive effect in the three-year analysis in comparison to individual years

(c) For main effect. “0” corresponds to values smaller than 0.01 or negative values obtained due to the computation method based on accumulated analysis of variance

Abbreviation: n.s.—not significant

(d) Defined as continuous regions of overlapping intervals ± 1.8 cM around QTL position (1.8 cM being half of the average length of two LOD support interval computed for all QTLs, see Xu, 2010).

Twelve QTLs were detected for length of main stem (LSt), and they were found to be unevenly distributed between populations (Table 4). Most QTLs had stable effects: out of 12 detected QTLs, interaction with the environment was found only for three (on 2H, 4H, and 5H.2). Among the detected QTLs prevailed these with positive effects for main stem and only four QTLs with negative effects contributed by Syrian genotypes were recorded: on 2H at Bmac0093 (QLSt.GH-2H), on 3H.1 at GBM1090 (QLSt.GH-3H.1-2), and on 7H.2 at SNP 6353–524 (QLSt.GH-7H.2) with Harmal alleles reducing stem length, and one on 5H.3 at SNP 4795–782 (QLSt.MC-5H.3) with CamB allele reducing stem length. These QTLs explained a relatively small proportion of phenotypic variability—from 4.01% to 11.29%. QLSt.MC-2H, found in the MCam population, with CamB allele increasing stem length, was located at SNP 5880–2547. This QTL had a major effect which was significant in 2011 and 2013 and explained 81.15% and 23.06% of phenotypic variation in the respective years. Three QTLs with positive and stable effects on stem length were localised on 3H.1—one in the GH population at SNP 5657–1187 (QLSt.GH-3H.1-1), one in MCam at SNP 5260–462 (QLSt.MC-3H.1), and one in LCam some 1.2 cM from SNP 4026–655 (QLSt.LC-3H.1). QLSt.GH-3H.1-1 with Harmal allele increasing stem length by 3.25 cm, explained in GH population 29.93% and 25.11% of variation in 2012 and 2013, respectively. QTLs in MCam and LCam were located in the same region (H) close to each other; QLSt.MC-3H.1 at position 105.75 cM explained a large proportion of the phenotypic variance in MCam, 96.66%, 34.36%, and 37.02% in subsequent years, whereas QLSt.LC-3H.1 at position 106.62, found in LCam, explained 37.47%, 9.41%, and 10.68% of variation in 2011, 2012, and 2013, respectively (Table 4).

Twelve QTLs were detected for length of main spike (LSp) (Table 4). Out of these QTLs, six were localised on chromosome 2H, the main QTLs being in region B at SNP 5880–2547 and in region F at SSR marker Bmag0720. All QTLs detected on 2H, except QLSp.MC-2H-2, were with alleles of Syrian genotype contributing to reduction in spike length. The main QTL detected in the MCam population (QLSp.MC-2H-1), positioned at 10.74 cM, explained 7.57% of variation in 2011, 29.42% in 2012, and 31.72% in 2013. The main QTL found in the GH population (QLSp.GH-2H-2), positioned at 55.72 cM, had a stable effect and explained 53.15% and 37.92% of phenotypic variation in subsequent years. QLSp.MC-2H-3, on 2H at position 106.85 cM, with a negative but stable over environments effect of CamB allele, explained 14.49%, 10.48%, and 8.23% of phenotypic variation in 2011, 2012, and 2013, respectively. CamB alleles decreasing spike length were also detected in QTLs localised in the linkage group 5H.3 in region K—in the MCam population (QLSp.MC-5H.3) some 1.02 cM from the SNP 314–559, and in LCam (QLSp.LC-5H.3) at the marker ConsensusGBS0138-2. Both QTLs explained 9.40% to 23.23% of phenotypic variation—higher values being noted in 2011. Among 12 QTLs, four were associated with significant increase of LSp contributed by CamB alleles, detected in the MCam population: QLSp.MC-1H.2, QLSp.MC-2H-2, QLSp.MC-3H.1, and QLSp.MC-7H.3. Among them three QTLs demonstrated stable effects over years and explained 6.34% to 19.24% of phenotypic variance (Table 4).

Twelve QTLs were detected for number of grains per main spike and 11 of them showed stable effects over years (Table 4). All QTLs located on 2H as well as those on 6H and 7H.2 showed negative effects on the number of grains per spike contributed by CamB or Harmal alleles. The main QTL was QNGS.MC-2H on 2H in region B, found in the MCam population at SNP 5880–2547, that explained from 71.73% to 83.98% of variation. At the same position QNGS.LC-2H-1 was detected in the LCam population, but its effect and percentage of explained variation were considerably lower. The main QTL detected in the GH population (QNGS.GH-2H-2), associated with negative effects of the Harmal allele, was located on 2H (region F) and explained 43.98% and 41.97% of variance in 2011 and 2012, respectively. Out of 12 QTLs, three QTLs, detected in the MCam population and located on 1H.1, 3H.1, and 4H, showed positive effects of CamB alleles, and one detected in GH on 1H.2 showed positive s of Harmal alleles. All four QTLs explained from 4.47% to 15.77% of phenotypic variation.

For grain weight per spike 12 QTLs were found, of which five were detected on chromosome 2H and one each in the linkage groups 1H.1, 3H.1, 3H.2, 4H, 5H.1, 7H.2, and 7H.3 (Table 4). Two QTLs (QGWS.MC-2H-1 and QGWS.LC-2H) were identified in both MCam and LCam populations on 2H in region B at SNP 5880–2547. They explained more than 50% of phenotypic variation in MCam and 16.25–27.58% in LCam. In both the populations, the effects of these QTLs were stable over environments, but in LCam they were considerably lower. On this chromosome important effects were realised by QGWS.GH-2H-2 identified in the GH population at SSR marker Bmag0720 and QGWS.MC-2H-2 identified in the MCam population at SNP 2580–1456. All these QTLs were with CamB or Harmal alleles reducing GWS. Two QTLs with positive effects of CamB alleles were found in the MCam population: on 4H in region I (QGWS.MC-4H) and 5H.1 in region J (QGWS.MC-5H.1). These two QTLs had stable effects over years and explained 13.01–14.75% and 7.32–8.30% of phenotypic variation, respectively.

Twenty-one QTLs for TGW were identified which were almost evenly distributed among populations: 7 in MCam, 6 in LCam, and 8 in GH (Table 4). Out of seven QTLs detected in MCam, three showed significant interaction with environments. The most significant was QTGW.MC-2H-1 mapped in marker GBM1214, where the CamB allele significantly reduced TGW. Other QTLs with stable effects of the CamB allele significantly decreasing TGW were found on 2H (QTGW.MC-2H-2) and in the linkage group 5H.3 (QTGW.MC-5H.3-1); however, the percentage of variance explained by this QTL was low (4.30–7.64%). Three QTLs with positive effects of CamB alleles were detected: on 3H.1 (QTGW.MC-3H.1), 5H.3 (QTGW.MC-5H.3-2), and 7H.3 (QTGW.MC-7H.3). Among six QTLs for TGW detected in the LCam population QTGW.LC-2H-1 on 2H was mapped in the same region (B) as in MCam, but its effect was significant only in 2011. Negative, stable effects of the CamB allele were also found on 4H (QTGW.LC-4H) at the marker ABC09432-1-1-160, which explained 12.55 to 14.35% of variance. QTLs with stable and positive effects of CamB alleles were revealed on 6H (QTGW.LC-6H) and in the linkage group 7H.2 (region L). QTL on 6H explained 14.16 to 16.19% of variance, while that on 7H.2 explained only about 8% of variance. Among QTLs detected for TGW in GH population the most significant were QTGW.GH-2H located at SNP 2822–739 and QTGW.GH-6H at the SSR marker HVM31, both with Harmal alleles reducing TGW. Out of the QTLs detected on other chromosomes only that on 4H (QTGW.GH-4H) had positive and stable additive effects of Harmal allele and explained 10.71% and 12.45% of variation in 2012 and 2013, respectively (Table 4).

Five QTLs for GY were detected in the analysed populations (Table 4): three in LCam, MCam, and GH on chromosome 2H at positions 10.74, 16.01, and 49.79 cM, respectively; one in LCam in the linkage group 3H.1; and one in the GH population in the linkage group 7H.2. All had the alleles from Syrian parental cultivars decreasing grain yield. No significant additive QTL effects were found in 2011. QTLs (QGY.LC-2H and QGY.MC-2H) on the chromosome 2H in the LCam and MCam populations were located at the SNP marker 5880–2547 and the SSR marker Bmag0692, respectively. Both these QTLs showed interaction with environments, and their effects were significant only in 2012 and explained a large proportion of phenotypic variation: 43.16% in LCam and 25.84% in MCam. QTLs detected in the GH population had stable effects: QGY.GH-2H located on the 2H chromosome (region E) showed a strong effect both in 2012 and in 2013 and explained 16.12% and 30.57% of variation, respectively, whereas that on chromosome 7H.2 (QGY.GH-7H.2) explained 8.82% and 16.72% of phenotypic variation in 2012 and 2013, respectively.

The length of projections of QTL support intervals onto the genomic sequence varied from 101 kbp to 299 Mbp. For interpretation we used only annotation of genes contained in the 73 intervals shorter than 20 Mbp as very long intervals result from serious distortion of the colinearity of SNPs in the linkage map and in the genomic sequence. The number of genes located in the considered intervals was 2260 (from 0 to 187 for individual QTLs); no particular GO term was overrepresented in the corresponding set of annotations (BINGO tool in Cytoscape, hypergeometric test at P = 0.05 with Bonferroni correction). The proportions of genes classified according to GO terms describing biological processes were different among traits and among QTL regions A-L (S3 Table). The term "lipid metabolic process" was overrepresented in the QTL regions for GY; the term "oxidation-reduction process"—for QTL region B. The proportions of annotations by molecular function GO terms also varied between traits and QTL regions significantly (results not shown).

### Stability of lines across environmental conditions

Stability analysis was performed for trait GY in MCam and LCam populations only because they were observed over three years, which is a minimum number of environments for the genotype environment (GE) and joint regression analysis (S4 Table). The analysis allowed for selection of two interesting subsets of lines: (1) stable lines (characterised by a non-significant GE interaction) that did not have a significant negative mean genotypic effect and (b) unstable lines of extensive character, that is, with a negative regression of genotypic effects on environmental effects. Stable lines provide relatively uniform and high yield across years; extensive lines behave especially well, in relation to other genotypes, in poor conditions (2011 in our case). Among stable and extensive lines we then selected those with alleles at marker 5880–2547 (linked closely to QTL for heading) inherited from CamB, and alleles at markers 5260–462 and 4026–655 (linked closely to QTL for stem length) inherited from Maresi and Lubuski, respectively. This gave the list of lines with genotypes that were favourable for early heading and short plant stature, but which did not have a significantly decreased grain yield: five stable lines in the MCam population (MCam053, MCam067, MCam075, MCam080, and MCam087), four in the LCam population (LCam057, LCam061, LCam077, and LCam087), and three extensive lines (LCam050, LCam071, and MCam071) (S4 Table).

## Discussion

In the present studies, three populations of RILs derived from crosses between European and Syrian cultivars were evaluated during a three-year experiment (2011 to 2013) conducted at Cerekwica in the Wielkopolska region of Poland. Huge differences in meteorological conditions between years were observed. A spring drought in 2011 resulted in a drastically low grain yield which was several times lower than that in 2012 and 2013. The main cause of the yield reduction in 2011 was the drying out of many plants before flowering, resulting in a reduction in the number of productive tillers per plot—among germinated seeds, less than 50% of plants evolved spikes with grains (data not shown). On the other hand, rainfall that occurred in late June and July resulted in good grain filling, especially in late heading genotypes, which were observed to have a higher number and weight of grains per main spike than early heading. It should be noted that these traits were observed in normally developed spikes (spikes that did not emerge from the sheathing leaf were not selected for measurement). In the years 2012 and 2013, more favourable conditions occurred for the barley crop in spring, particularly in the first 10-day period in May, with precipitation amounting to, respectively, 133.5% and 343.9% of the long-term average.

Large differences in the weather conditions between 2011 and the next two years (2012 and 2013) resulted in significant differences in mean values of observed traits between years as well in the occurrence of genotype × environment interaction, especially for grain yield. Thus the experiments turned out to be suitable for characterisation of the genotype-phenotype associations for a broad range of expression of traits.

### Genetic map

In the present study, a consensus map was constructed from a total of 819 polymorphic SSR and SNP markers from the individual maps of the three RIL populations. Segregation distortions were observed in all three populations, and most of the markers that exhibited the distorted segregations were gathered in clusters and skewed towards the European parental cultivars. Regions of distorted segregation have been reported in many crop species, both in natural and in mapping populations [7,47]. A high level of distorted segregation ratios in this study might be partially related to the type of mapping population and the diverse pedigree of the parental lines [48]. A high percentage of markers with distorted segregation ratios in an RIL population might be caused by environmental and artificial selection in favour of unknown genetic factors acting over several generations during its development [49]. We also noted that the order of populations according to the fraction of markers with a distorted segregation, from the lowest in GH to the highest in LCam, corresponded to their ordering according to the similarity (simple matching) coefficient between parental cultivars computed from the marker data: 0.59, 0.61, 0.64 for GH, MCam, and LCam, respectively.

The total length of the consensus map constructed in the present studies was 953.8 cM, with the average interval between the neighbouring loci being 1.2 cM. The map consisted of 13 linkage groups, which were formed at high LOD scores (6 to 11) to avoid false positive linkages among distorted markers and to ensure the correct locus order and genetic distances among large numbers of markers within the groups. In general, the marker order in linkage groups agreed with previously reported barley maps containing both SSR and SNP markers [22,23] except for some inconsistencies at the centromeric regions of the chromosomes, and the groups provided good barley genome coverage. In the literature it has been suggested that linkage analysis with the use of a large number of markers may lead to false positive linkages even with a LOD score of six. The use of higher LOD threshold values prevents markers from different chromosomes being incorrectly assigned to the same linkage group but it may result in the chromosomes being split into fragmented linkage groups [7].

### QTLs for yield-forming traits

All the QTLs found for grain yield in LCam and MCam populations examined for three years showed interaction with environments, but in the GH population, which was examined only for two years with similar and desirable conditions for plant development, this interaction was not significant. In the dry year 2011, no QTL effect for grain yield appeared to be significant. This could not be explained by the fact that small effects observed in 2011 were analysed jointly with the much larger effects observed in 2012 and 2013. The same conclusion could be drawn by comparison of additive effects and means for grain yield: in 2011 the additive effects amounted to 1 to 3% of the mean grain yield, but in 2012 and 2013 to 7 to 14%. Thus, the detection of QTL for yield in extremely unfavourable conditions may not be possible.

Out of five QTLs for GY found in the present studies, three were located on the 2H chromosome. In LCam, QTLs for HS, GWS, NGS, GY and TGW were detected at the same position of 10.74 cM. In MCam, QGY.MC-2H was located at Bmag0692 at position 16.01 cM. This marker was mapped on chromosome 2H near marker GBMS229, which was reported by Li et al. [14] as associated both with grain yield and with earliness. According to Varshney et al. [22], GBMS229 was located at the same position as GBM1214, at which, in the present study, the QTLs for TGW (QTGW.MC-2H-1) and days to heading (QHS.MC-2H) were detected.

The number of QTLs associated with grain yield detected in other studies varied, depending on the studied populations and environmental conditions. Peighambari et al. [49] found only one QTL for grain yield on chromosome 2H. However, in their study QTLs for yield components were detected also on chromosomes 1H and 5H. Three QTLs for grain yield were detected by Comandran et al. [50]. One of those QTLs was located in the centromeric region of chromosome 2H and the other two were detected on the long arm of chromosome 7H; these could correspond to the three QTLs found on 2H and the one found on 7H.2 in our study. Mansour et al. [7] reported four QTLs for grain yield located on chromosomes 1H, 2H.1, 5H.3, and 7H, but they did not find any association between grain yield and earliness in the region of SNP 11_21015. This result is in contrast to our study, where QTL for GY was detected on 2H at this SNP. Mansour et al. [7] found the most significant QTL for grain yield located in linkage group 5H.3, at the *Vrn-H1* locus.

Differences between the results of the current experiments and those published by Mansour et al. [7] may be caused by the different growth habit of studied lines on the one hand, and various climatic conditions, in which experiments were conducted, on the other. Barley lines examined in our studies were of spring growth habit, whereas those by Mansour et al. [7] were of facultative or winter growth habit with a mild and high vernalisation requirements, respectively. In present experiments lines were sown in spring and plants developed under increasing day length—from 12.7 hs in April (sowing) to about 16 hs in June (flowering), whereas in experiments conducted by Mansour et al. [7] trials were sown in autumn and during experiments day length varied from about 9 to 13–14 hours from sowing to flowering. Thus, in our study the day length had greater effect on grain yield than vernalisation requirements, while in the studies conducted by Mansour et al. [7] vernalisation requirements prevailed.

In the present studies, 15 QTLs related to heading date were detected. A substantial proportion of these QTLs are consistent with previously identified QTLs in various studies [8,13,51–56]. Some of these QTLs were located in genomic regions that were previously reported to harbour genes involved in flowering time regulation. The six QTLs controlling the HS were detected on chromosome 2H. QTLs QHS.LC-2H-1 and QHS.GH-2H-1 at SNP 5880–2547 had major effects on both LCam and GH populations, as did QHS.MC-2H located at GBM1214 in MCam. According to Muñoz-Amatriaín et al. [57], the SNP marker 5880–2547 (11_21015) maps close to the BOPA2 markers 12_30871 and 12_30872, which are SNPs in *Ppd-H1* gene, considered as the major photoperiod response locus [53]. Mansour et al. [7] reported three QTLs for heading date on chromosomes 2H, 5H.3, and 7H; moreover, the QTL on 2H was also located at SNP 11_21015, which is consistent with our results. Comadran et al. [50] reported five QTLs for heading date located on chromosomes 1H, 2H, 3H, and 5H. Their research revealed that the largest effects were exhibited by two QTLs detected in the centromeric region of 2H, where a major gene affecting heading (*eam6*) was previously reported [5]. Ren et al. [9] identified three QTLs determining heading date on chromosomes 2H, 3H, and 7H. The authors suggested that QTL on 3H is the same as the QTL for heading previously reported by Teulat et al. [10] and von Korff et al. [12]. Two QTLs controlling HS were detected in our study in region K on 5H.3, which is in accordance with the results reported by Thomas et al. [54] and Marquez-Cedillo et al. [58]. In the MCam population, the QTL conferring heading was detected in the 3H.1 linkage group at the position of 102.19 cM, 3.56 cM from the QTL affecting the stem length. QTLs for LSt on 3H.1 detected in our study for MCam and LCam populations were found close to each other, at positions 105.75 cM (QLSt.MC-3H.1) and 106.62 cM (QLSt.LC-3H.1), respectively. The QLSt.MC-3H.1 was linked to the SNP 5260–462 (11_10754) and at 0.45 cM from the SNP 6716–823 (11_10867), which has been suggested as corresponding to the *sdw1/denso* locus [59]. In the pedigree of cv. Maresi, which was used to generate the MCam population, the cv. Diamant (an X-ray mutant of the cv. Valticky) is said to carry the *sdw1/denso* gene (http://genbank.vurv.cz/barley/pedigree/) [60]. The main phenotypic effect of this gene is the decrease in plant height. This is in agreement with our observation that the Maresi allele reduced the stem length by approximately 10 cm. In addition to the reduced plant height, semidwarf *sdw1/denso* mutants were characterised by an increased time to heading, late maturity, decreased 1000-grain weight, and decreased grain weight [61–65]. A similar result was obtained by Ponce-Molina et al. [66] who found the Beatrix allele on that region of 3H associated with reduced plant height, later heading and decreased TGW. This agrees with the effects of QTLs detected in our studies in region H; decreased TGW and delayed HS were associated with the allele contributed by Maresi. The QTL for LSt detected in the 2H linkage group in MCam (QLSt.MC-2H) was linked to SNP 5880–2547 (11_21015) and overlapped with the QTL hot-spot (region B). This corresponds to the results of Mansour et al. [7], who mapped one of the five detected QTLs for plant height on 2H.1, between *Ppd-H1* and SNP 11_21015. Thus, our results confirm the association between earliness and plant height reported by several authors [12,67–68]. Jia et al. [69] proposed GA-20 oxidase as a candidate for barley *sdw1/denso* gene. GA-20 oxidase is involved in the gibberellin signalling pathway, which affects plant growth and development [70].

Out of 21 QTLs for TGW detected, only one was found in the same region (B on 2H) in LCam and MCam populations. Li et al. [6] identified a QTL for the 1000-grain weight on chromosome 2H at Bmag0692 (which we mapped close to region B). Pillen et al. [8] reported 12 QTLs for TGW located on four chromosomes, and three of them on 2H, whereas Ren et al. [9] identified only two QTLs for 1000-grain weight—on chromosomes 2H (Bmag0518) and 7H (GMS46). In our studies, QTGW.MC-7H.3 was linked to EBmac0755. This SSR is reported to be associated with the QTL affecting yield [8]. In our studies, among the detected QTLs for TGW, the strongest were identified on 6H. This is in agreement with the results reported by Li et al. [6] and von Korff et al. [2] where the QTLs for the TGW located on 6H were the most significant. On the other hand, in the studies by Mansour et al. [7], the minor QTL for this trait was detected on 6H chromosome. They found the most significant QTL for TGW in their 4H.1 linkage group at SNP 11_10379. On the basis of the map reported by Szűcs et al. [71] and Muñoz-Amatriaín et al. [57], the authors conclude that this QTL was located in the same region as the QTLs for TGW described previously by Backes et al. [72] and Kjaer and Jensen [73].

The localisation of QTLs in our studies reflects the genetic differences between parental genotypes; QTLs for GH were situated in different regions of the chromosomes from the QTLs for the half-sibling populations LCam and MCam. We also noticed a small dissimilarity in the distribution of QTLs among individual regions even in half-sibling populations. For example, QTL for LSt (QLSt.MC-2H) in region B was detected only for MCam. QTLs associated with earliness were located in region B for all mapping populations, indicating that region B affected earliness regardless of the genetic background. In addition, this is the only case where all the three RIL populations used in our experiments had common QTLs for the same trait.

Alleles from Syrian parental cultivars contributed, in general, a consistent decrease in spike length, grain number, and weight per spike as well as grain yield. However, the analyses revealed that both Syrian cultivars contributed also alleles increasing time to heading and reducing stem length: QHS.MC-5H.3 and QHS.LC-5H.3 for later heading; and QLSt.MC-5H.3, QLSt.GH-2H, QLSt.GH-3H.1-2, and QLSt.GH-7H.2 for shorter stem. QHS-5H.3 may correspond to Vrn-H1, as detected by Rollins et al. [74] and Mansour et al. [7]. CamB and Harmal also contributed alleles increasing TGW; seven QTLs with positive effects for that trait were detected. QTLs with CamB alleles increasing spike length were found on 2H, 3H.1, and 7H.3; with alleles increasing number of grains in spike on 1H.1, 3H.1, and 4H; and with alleles increasing grain weight per spike on 4H and 5H.1.

### Functional annotation of QTLs

For a biological interpretation of the QTL regions identified on the basis of linkage analysis, we refer to the annotation of SNP using the Ensembl Plants barley gene space. Such interpretation must be treated as putative because of the biological variability in phenotyping experiments and a limited resolution of the linkage map. Despite this, we think that it is useful to exploit the current resources of genomic annotations for the purpose of QTL interpretation, and we did this using a method similar to the one implemented by Cantalpiedra et al. [75]. It should be noted that the mapping of the BOPA1 SNP sequences to barley Ensembl contigs does not give completely new information, as some of them were used to create a linear order of those contigs [31]. However, as a complete and updated resource containing all SNP-contig assignments is, to our knowledge, not available, we decided to do a remapping using the current version of Ensembl data. For a subset of SNP markers, we checked our mapping results against the Triticeae Toolbox database (triticeaetoolbox.org, mappings to NCBI database) and MIPS data (mappings to FPC contigs, file ‘experimental_marker.txt’); generally, an agreement of results was found, although in some cases our blast to Ensembl sequence gave a better result in a different contig or position. A full comparison of our SNP mapping results with those existing in the databases is, however, beyond the scope of this paper.

The detected overrepresentation of genes annotated by the GO term “lipid metabolic process” in the QTL regions determining GY sounds reliably due to the essential role of lipids in all plant cells. Lipids, required for membrane biogenesis, are also considered as an important form of stored carbon and energy, which can be utilized for grain yield improvement [76]. The discussion about the overrepresentation of genes related to “oxidation-reduction process” found in region B seems to be a wide issue. Redox reactions are the basis of many processes related to photosynthesis or respiration, thus this overrepresentation may be the reason why this interval of 2HS has been widely reported in literature to be a QTL-rich region. Generally, the predominant role of the *Ppd-H1* locus in this hot-spot region is suggested [53]; this locus was formally located outside of QTL support intervals used in our functional annotation, but its role in shaping the variation observed in our experiments is very likely.

The annotation of QTL regions A-L by genes occurring in the projected support intervals showed the presence of a number of genes with functions described in Ensembl Plants (among them, 45 genes with defined transcript names). Region H in the chromosome 3HL aroused our curiosity due to the co-localisation of QTLs for LSt and HS. As previously mentioned, this region corresponds to the *sdw1/denso* locus. QTL annotation of region H revealed the presence of MLOC_56462 which is considered as the *Hv20ox2* gene [77]. Until now, it has been interpreted as the main candidate gene in 3HL chromosome affecting plant height in barley [64]. However, in this study, another gibberellin oxidase 20 in this interval has been identified–*GA20ox3* (MLOC_66389). The two genes are paralogues according to Ensembl Plants database. The function of the *GA20ox3* gene has not been proven so far in barley. Qin et al. [78] demonstrated the dual role of the *GA20ox3* in rice–plant stature regulation and participation in response to pathogen infection. Based on the *GA20ox3* expression profile authors assumed that it can complement the function of the homologous genes *GA20ox1* and *GA20ox2* in gibberellin biosynthesis in rice.

In region J (containing QTL for HS), it is interesting to note the presence of MLOC_824, to which several transcripts of the phytochrome C gene were assigned (*PHYC*, *PHYC-210*, *PHYC-211*, *PHYC-212*, *PHYC-213*, and *PHYC-214*). Plants can monitor almost all facets of light, such as direction, duration, quantity, and wavelength by using photoreceptors. Phytochromes are dimeric proteins that function as red and far-red light sensors influencing nearly every phase of the plant life cycle. These proteins are encoded by three genes (*PHYA*, *PHYB*, and *PHYC*) in most monocots [79]. In barley, the chromosome region encompassing PHYC gene has been associated with differences in flowering time under inductive LD photoperiods [80]. *PHYC* is tightly linked to the vernalization gene *Vrn-H1* [81], previously mentioned in relation to QHS-5H.3.

The QTL annotation by the projected support intervals revealed one of the major flowering time gene–*Vrn-H3* (MLOC_68576) in region L, linked in our study to four QTLs identified in GH population (QLSp.GH-7H.2, QGWS.GH-7H.2, QHS.GH-7H.2, Q.LSt.GH-7H.2). The candidate for *Vrn-H3*, *HvFT1*, has been recently identified, with evidences pointing at an important role in the integration of the vernalization and photoperiod pathways. In barley, expression of *HvFT1* is induced by long day conditions and promotes flowering [82]. Barley genotypes with a photoperiod responsive *Ppd-H1* allele are characterized by elevated expression of *Vrn-H3* (*HvFT1*) homologous to the Arabidopsis gene *FLOWERING LOCUS T (FT)* [83]. It is noteworthy that one of the identified four mentioned QTLs was referred to heading stage (HS). Although the spring barley does not require vernalization in order to flower, the vernalisation and photoperiod pathways correspond to each other to promote flowering in many crop species [84].

The genes mentioned above can be putatively considered as candidates for being a part of the polygenic model for the observed traits. However, we cannot exclude that other genes located in the QTL regions have such a role, and may also be responsible for reaction of plants to the environment. We think that a lack of overrepresentation of GO terms for genes found in all QTL intervals that we observed was to be expected, as multiple molecular networks, including signal transduction, enzyme activity, energy distribution, ion transport and the activities of transcription factors are involved in the traits' expression and stress responses.

## Conclusions

In conclusion, the phenomic and molecular approaches permitted us to distinguish the lines combining desirable features or alleles from their parents, that is, early heading from CamB and short plant stature from Maresi, but with an acceptable grain yield. Numerous outcomes of our linkage analysis confirmed the previous results on yield-related QTLs in spring barley. However, the localisation of a large number of QTLs near major genes, determining earliness and plant height, clearly indicates a larger role of these genes in the development of traits associated with grain yield. This provides new information on the genetic determination of quantitative traits in general. Both stable and extensive lines could be distinguished by stability analysis in the half-sibling populations MCam and LCam. Therefore, several of our results are significant for breeders, considering that they indicate the possibility of selecting genotypes that combine acceptable yield with advantageous characteristics contributed by the Syrian cultivars. It may be hypothesised that the lines which were stable in our experiments conducted in years of different weather conditions will prove to be more resistant to drought. This proposal needs to be confirmed by additional, appropriate experiments with application of water-deficit stress in controlled conditions.

## Supporting Information

S1 Fig A consensus linkage map of barley constructed using three RIL mapping populations. (TIF)

S1 Table Ten-day values of air temperature, water vapor pressure deficit, precipitation (P), evapotranspiration (ETR), and ETR–P during vegetation period in 2011–2013. (DOCX)

S2 Table Summary statistics for traits observed for parental cultivars and RIL populations. (DOCX)

S3 Table Classification of barley genes located in genomic regions corresponding to LOD support intervals for all phenotypic traits according to their GO annotation. (DOCX)

S4 Table Results of stability analysis for RIL populations MCam and LCam. (DOCX)
