# Supplementary figures and images for: Quantitative Trait Loci for Yield and Yield-Related Traits in Spring Barley Populations Derived from Crosses between European and Syrian Cultivars
Source: PLoS One. 2016 May 26;11(5):e0155938. doi: 10.1371/journal.pone.0155938 (PMC4881963; doi:10.1371/journal.pone.0155938)

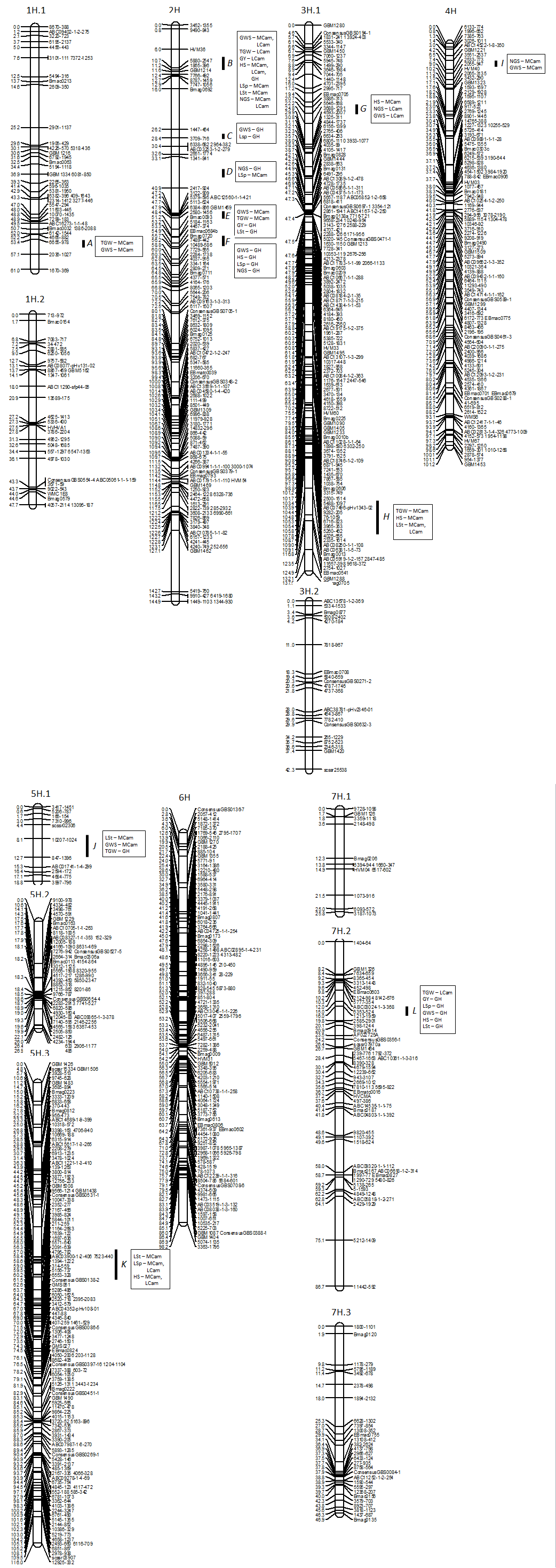

Supplement: S1 Fig — (TIF) [file pone.0155938.s001.tif]
