# Supplementary material for: Quantitative Trait Loci for Yield and Yield-Related Traits in Spring Barley Populations Derived from Crosses between European and Syrian Cultivars
Source: PLoS One. 2016 May 26;11(5):e0155938. doi: 10.1371/journal.pone.0155938 (PMC4881963; doi:10.1371/journal.pone.0155938)
Supplement: S1 Table — (DOCX) [file pone.0155938.s002.docx]

S1 Table. Ten-day values of air temperature, water vapor pressure deficit, precipitation (P), evapotranspiration (ETR), and ETR–P during vegetation period in 2011–2013

| Year | April | | | Month | May | | | Month | June | | | Month | July | | | Month | Mean |
| --- | --- | --- | --- | --- | --- | --- | --- | --- | --- | --- | --- | --- | --- | --- | --- | --- | --- |
|  | 1 | 2 | 3 |  | 1 | 2 | 3 |  | 1 | 2 | 3 |  | 1 | 2 | 3 |  | IV–VII |
| Air temperature [°C] | | | | | | | | | | | | | | | | | |
| 2011 | 10.5 | 8.9 | 13.1 | 10.8 | 9.4 | 14.6 | 15.7 | 13.2 | 17.9 | 16.9 | 17.2 | 17.32 | 17.2 | 19.3 | 15.3 | 17.3 | 14.65 |
| 2012 | 4.6 | 7.4 | 14.5 | 8.8 | 14.8 | 12.4 | 17.0 | 14.7 | 13.3 | 16.8 | 17.2 | 15.75 | 21.1 | 15.9 | 19.0 | 18.7 | 14.50 |
| 2013 | 1.5 | 11.3 | 11.9 | 8.3 | 14.8 | 15.7 | 12.4 | 14.3 | 16.0 | 19.2 | 16.2 | 17.15 | 18.9 | 17.4 | 20.8 | 19.0 | 14.68 |
| Water vapor pressure deficit [hPa] | | | | | | | | | | | | | | | | | |
| 2011 | 4.2 | 3.7 | 5.8 | 4.6 | 5.9 | 6.3 | 6.7 | 6.3 | 7.0 | 7.3 | 7.3 | 7.2 | 6.6 | 7.3 | 4.1 | 6.0 | 6.02 |
| 2012 | 3.6 | 3.9 | 9.0 | 5.5 | 6.8 | 6.4 | 9.8 | 7.7 | 4.9 | 5.9 | 6.0 | 5.6 | 6.7 | 4.5 | 7.8 | 6.3 | 6.27 |
| 2013 | 2.1 | 5.4 | 6.0 | 4.5 | 6.4 | 6.6 | 3.9 | 5.6 | 4.5 | 9.4 | 5.9 | 6.6 | 7.6 | 6.4 | 9.9 | 7.9 | 6.16 |
|  | Precipitation [mm] | | | | | | | | | | | | | | | |  |
| 2011 | 4.3 | 1.7 | 1.6 | 7.6 | 0.7 | 31.2 | 6.8 | 38.6 | 26.7 | 16.2 | 35.6 | 78.5 | 33.5 | 67.1 | 34.6 | 135.2 | 259.8 |
| 2012 | 1.9 | 2.5 | 7.7 | 12.1 | 23.1 | 21.8 | 0.1 | 44.9 | 11.7 | 39.3 | 20.4 | 71.5 | 69.6 | 60.8 | 12.7 | 143.1 | 271.6 |
| 2013 | 0.0 | 13.1 | 6.8 | 19.9 | 59.5 | 3.7 | 39.2 | 102.4 | 25.3 | 0.1 | 72.5 | 97.8 | 4.4 | 4.7 | 44.0 | 53.0 | 273.1 |
| Norm | 12.8 | 14.3 | 15.9 | 43.1 | 17.3 | 18.7 | 19.9 | 56.0 | 21.2 | 22.7 | 24.3 | 68.2 | 26.0 | 27.4 | 28.1 | 81.5 | 248.8 |
| Long-term average values of ten-day ETR | | | | | | | | | | | | | | | | | |
|  | 11.4 | 12.8 | 15.4 | 39.6 | 19.5 | 24.9 | 29.7 | 74.1 | 33.8 | 35.9 | 36.4 | 106.0 | 36.2 | 33.8 | 30.0 | 100.0 | 319.8 |
|  | ETR – Precipitation | | | | | | | | | | | | | | | | |
| 2011 | 7.1 | 11.1 | 13.9 | 32.1 | 18.8 | −6.3 | 22.9 | 35.5 | 7.1 | 19.7 | 0.9 | 27.6 | 2.7 | −33.3 | −4.6 | −35.2 | 60.0 |
| 2012 | 9.5 | 10.3 | 7.7 | 27.5 | −3.6 | 3.1 | 29.6 | 29.1 | 22.1 | −3.5 | 16.0 | 34.6 | −33.4 | −27.1 | 17.4 | −43.1 | 48.2 |
| 2013 | 11.4 | −0.3 | 8.6 | 19.8 | −40.0 | 21.2 | −9.5 | −28.3 | 8.5 | 35.8 | −36.0 | 8.2 | 31.7 | 29.1 | −13.9 | 47.0 | 46.7 |
