# Supplementary material for: Quantitative Trait Loci for Yield and Yield-Related Traits in Spring Barley Populations Derived from Crosses between European and Syrian Cultivars
Source: PLoS One. 2016 May 26;11(5):e0155938. doi: 10.1371/journal.pone.0155938 (PMC4881963; doi:10.1371/journal.pone.0155938)
Supplement: S2 Table — (DOCX) [file pone.0155938.s003.docx]

S2 Table. Summary statistics for traits observed for parental cultivars and RIL populations MCam, LCam

and GH in 2011–2013 (s.e. – standard error of mean value; c.v. – coefficient of variation)

| Trait | Population | Year | Parent* | | | | Recombinant inbred lines | | | | |
| --- | --- | --- | --- | --- | --- | --- | --- | --- | --- | --- | --- |
|  |  |  | P1 | | P2 | |  |  |  |  |  |
|  |  |  | mean | s.e. | mean | s.e. | mean | s.e. | min | max | c.v. |
| Heading stage  (days) | MCam | 2011 | 54.00 | 0.00 | 45.67 | 0.33 | 53.43 | 0.32 | 45.67 | 57.33 | 6.08 |
|  |  | 2012 | 48.33 | 0.33 | 44.00 | 0.00 | 46.86 | 0.29 | 41.00 | 51.33 | 6.28 |
|  |  | 2013 | 56.67 | 0.67 | 44.67 | 0.67 | 54.02 | 0.39 | 44.00 | 59.33 | 7.21 |
|  | LCam | 2011 | 53.67 | 0.33 | 45.67 | 0.33 | 52.78 | 0.32 | 46.00 | 57.33 | 6.10 |
|  |  | 2012 | 45.67 | 0.33 | 43.67 | 0.33 | 45.68 | 0.25 | 41.00 | 50.00 | 5.40 |
|  |  | 2013 | 54.67 | 1.67 | 44.67 | 0.67 | 53.56 | 0.30 | 47.00 | 58.67 | 5.67 |
|  | GH | 2012 | 47.67 | 0.33 | 40.33 | 0.33 | 46.08 | 0.16 | 41.33 | 49.33 | 3.56 |
|  |  | 2013 | 51.67 | 0.67 | 44.00 | 0.00 | 49.81 | 0.19 | 44.67 | 53.00 | 3.80 |
| Length of main stem  (cm) | MCam | 2011 | 52.00 | 1.53 | 50.67 | 2.40 | 47.29 | 0.53 | 35.33 | 65.67 | 11.14 |
|  |  | 2012 | 52.33 | 1.45 | 56.67 | 3.33 | 55.44 | 0.82 | 34.67 | 73.67 | 14.85 |
|  |  | 2013 | 58.33 | 3.84 | 68.67 | 1.33 | 56.35 | 0.80 | 43.00 | 76.67 | 14.23 |
|  | LCam | 2011 | 49.00 | 5.86 | 52.67 | 2.60 | 49.49 | 0.27 | 42.67 | 57.00 | 5.52 |
|  |  | 2012 | 69.00 | 2.08 | 52.67 | 2.67 | 64.86 | 0.53 | 50.67 | 81.00 | 8.17 |
|  |  | 2013 | 60.33 | 1.45 | 62.33 | 2.60 | 55.52 | 0.50 | 41.33 | 69.00 | 9.03 |
|  | GH | 2012 | 62.67 | 3.71 | 38.00 | 1.15 | 52.50 | 0.59 | 38.33 | 70.33 | 11.30 |
|  |  | 2013 | 58.67 | 3.28 | 47.33 | 2.33 | 55.50 | 0.65 | 44.00 | 74.33 | 11.64 |
| Length of main spike (cm) | MCam | 2011 | 6.73 | 0.19 | 6.87 | 0.13 | 6.90 | 0.07 | 5.07 | 8.53 | 10.03 |
|  |  | 2012 | 7.60 | 0.26 | 6.17 | 0.22 | 6.98 | 0.08 | 5.33 | 9.00 | 11.55 |
|  |  | 2013 | 8.03 | 0.22 | 6.57 | 0.20 | 7.83 | 0.09 | 5.60 | 10.17 | 11.58 |
|  | LCam | 2011 | 6.83 | 0.52 | 5.93 | 0.09 | 6.79 | 0.05 | 5.80 | 8.10 | 7.67 |
|  |  | 2012 | 7.27 | 0.63 | 6.60 | 0.40 | 7.39 | 0.06 | 6.20 | 9.40 | 8.37 |
|  |  | 2013 | 8.03 | 0.64 | 7.00 | 0.06 | 7.65 | 0.07 | 5.87 | 9.70 | 9.60 |
|  | GH | 2012 | 8.13 | 0.09 | 4.83 | 0.58 | 6.97 | 0.08 | 5.00 | 8.87 | 12.19 |
|  |  | 2013 | 8.40 | 0.38 | 5.73 | 0.70 | 6.98 | 0.10 | 4.80 | 9.57 | 14.37 |
| Number of grains per main spike | MCam | 2011 | 18.83 | 0.75 | 15.82 | 1.53 | 18.96 | 0.24 | 10.87 | 24.38 | 12.58 |
|  |  | 2012 | 19.97 | 1.13 | 15.67 | 0.38 | 18.96 | 0.26 | 11.03 | 24.80 | 13.73 |
|  |  | 2013 | 23.10 | 0.53 | 17.77 | 0.58 | 22.32 | 0.25 | 16.03 | 28.93 | 10.99 |
|  | LCam | 2011 | 18.63 | 0.55 | 13.60 | 0.52 | 17.50 | 0.18 | 13.08 | 21.63 | 10.51 |
|  |  | 2012 | 20.33 | 1.53 | 16.10 | 0.32 | 20.48 | 0.24 | 14.07 | 25.83 | 11.88 |
|  |  | 2013 | 22.40 | 0.75 | 16.63 | 1.35 | 20.84 | 0.22 | 14.70 | 25.33 | 10.43 |
|  | GH | 2012 | 20.17 | 0.73 | 8.87 | 2.55 | 16.16 | 0.26 | 10.70 | 22.90 | 16.21 |
|  |  | 2013 | 22.37 | 0.84 | 13.60 | 1.90 | 18.09 | 0.27 | 11.80 | 23.90 | 14.81 |
| Grain weight per main spike (g) | MCam | 2011 | 0.89 | 0.05 | 0.76 | 0.13 | 0.94 | 0.02 | 0.56 | 1.32 | 16.52 |
|  |  | 2012 | 0.98 | 0.10 | 0.77 | 0.02 | 0.95 | 0.02 | 0.61 | 1.33 | 16.18 |
|  |  | 2013 | 1.14 | 0.03 | 0.79 | 0.00 | 1.12 | 0.02 | 0.76 | 1.59 | 14.39 |
|  | LCam | 2011 | 0.91 | 0.05 | 0.57 | 0.04 | 0.86 | 0.01 | 0.58 | 1.10 | 13.37 |
|  |  | 2012 | 1.00 | 0.12 | 0.77 | 0.03 | 1.06 | 0.02 | 0.74 | 1.41 | 14.14 |
|  |  | 2013 | 1.03 | 0.03 | 0.75 | 0.06 | 0.99 | 0.01 | 0.72 | 1.25 | 12.44 |
|  | GH | 2012 | 1.07 | 0.09 | 0.45 | 0.15 | 0.89 | 0.02 | 0.53 | 1.22 | 18.11 |
|  |  | 2013 | 1.17 | 0.04 | 0.65 | 0.12 | 0.91 | 0.02 | 0.51 | 1.25 | 16.77 |
| 1000-grain weight  (g) | MCam | 2011 | 46.98 | 0.97 | 47.40 | 3.30 | 49.46 | 0.31 | 40.78 | 58.21 | 6.22 |
|  |  | 2012 | 49.12 | 2.84 | 49.20 | 1.06 | 49.88 | 0.35 | 41.46 | 57.67 | 7.05 |
|  |  | 2013 | 49.15 | 0.34 | 44.86 | 1.51 | 50.12 | 0.28 | 43.72 | 59.53 | 5.52 |
|  | LCam | 2011 | 48.76 | 1.57 | 41.92 | 1.37 | 48.94 | 0.30 | 40.83 | 55.60 | 6.14 |
|  |  | 2012 | 49.13 | 1.82 | 47.95 | 1.02 | 51.83 | 0.30 | 44.70 | 58.46 | 5.74 |
|  |  | 2013 | 46.19 | 0.47 | 45.08 | 0.29 | 47.36 | 0.32 | 39.27 | 53.82 | 6.81 |
|  | GH | 2012 | 52.80 | 2.39 | 50.62 | 2.71 | 54.93 | 0.38 | 45.29 | 65.31 | 6.98 |
|  |  | 2013 | 52.35 | 1.41 | 47.41 | 2.84 | 50.16 | 0.36 | 41.48 | 57.94 | 7.11 |
| Grain yield (g) | MCam | 2011 | 130.00 | 26.34 | 115.00 | 40.93 | 116.12 | 2.00 | 64.17 | 161.67 | 17.21 |
|  |  | 2012 | 593.33 | 64.31 | 368.33 | 19.22 | 657.29 | 13.22 | 296.67 | 945.00 | 20.12 |
|  |  | 2013 | 576.67 | 29.49 | 380.00 | 5.77 | 562.59 | 8.83 | 383.33 | 843.33 | 15.70 |
|  | LCam | 2011 | 113.33 | 22.05 | 71.67 | 14.81 | 116.21 | 1.87 | 63.75 | 160.00 | 16.08 |
|  |  | 2012 | 658.33 | 44.75 | 283.33 | 12.02 | 636.68 | 13.40 | 251.67 | 925.00 | 21.05 |
|  |  | 2013 | 553.33 | 48.07 | 440.00 | 17.32 | 520.40 | 12.20 | 280.00 | 873.33 | 23.45 |
|  | GH | 2012 | 1018.33 | 55.25 | 358.33 | 36.55 | 690.38 | 12.03 | 390.00 | 961.67 | 17.42 |
|  |  | 2013 | 776.67 | 53.64 | 300.00 | 35.12 | 538.21 | 8.70 | 360.00 | 763.33 | 16.16 |

*Parent:

MCam: P1 – Maresi; P2 – CamB

LCam: P1 – Lubuski; P2 – CamB

GH: P1 – Georgia; P2 - Harmal
