# Supplementary material for: Quantitative Trait Loci for Yield and Yield-Related Traits in Spring Barley Populations Derived from Crosses between European and Syrian Cultivars
Source: PLoS One. 2016 May 26;11(5):e0155938. doi: 10.1371/journal.pone.0155938 (PMC4881963; doi:10.1371/journal.pone.0155938)
Supplement: S3 Table — (DOCX) [file pone.0155938.s004.docx]

S3 Table. Classification of barley genes located in ±200 kbp genomic regions around SNPs linked to QTLs identified for all phenotypic traits according to their GO annotation

| Gene ontology term | 1000-grain weight | Grain weight per m^2^ | Grain weight per main spike | Heading stage | Length of main spike | Length of main stem | Number of grains per main spike | Total |
| --- | --- | --- | --- | --- | --- | --- | --- | --- |
| Biological process | | | | | | | | |
| biological_process | 5 | 0 | 8 | 2 | 5 | 3 | 5 | 28 |
| intracellular protein transport | 3 | 1 | 2 | 2 | 2 | 3 | 2 | 15 |
| metabolic process | 6 | 1 | 4 | 1 | 1 | 4 | 5 | 22 |
| oxidation-reduction process | 10 | 0 | 1 | 3 | 0 | 3 | 2 | 19 |
| protein phosphorylation | 8 | 0 | 1 | 2 | 3 | 3 | 4 | 21 |
| regulation of transcription, DNA-templated | 6 | 1 | 1 | 2 | 1 | 3 | 2 | 16 |
| transmembrane transport | 1 | 0 | 3 | 1 | 3 | 4 | 2 | 14 |
| transport | 5 | 0 | 2 | 1 | 2 | 2 | 2 | 14 |
| Total | 44 | 3 | 22 | 14 | 17 | 25 | 24 | 149 |
| Cellular component | | | | | | | | |
| chloroplast | 8 | 1 | 3 | 3 | 2 | 4 | 0 | 21 |
| cytoplasm | 4 | 0 | 2 | 1 | 3 | 2 | 3 | 15 |
| cytosol | 1 | 0 | 3 | 0 | 2 | 2 | 4 | 12 |
| integral component of membrane | 5 | 0 | 3 | 1 | 5 | 5 | 3 | 22 |
| membrane | 8 | 1 | 4 | 0 | 6 | 9 | 4 | 32 |
| nucleus | 10 | 1 | 3 | 1 | 3 | 3 | 5 | 26 |
| Total | 36 | 3 | 18 | 6 | 21 | 25 | 19 | 128 |
| Molecular function | | | | | | | | |
| ATP binding | 14 | 1 | 3 | 3 | 9 | 8 | 9 | 47 |
| binding | 3 | 2 | 2 | 2 | 2 | 2 | 2 | 15 |
| catalytic activity | 7 | 1 | 3 | 4 | 2 | 4 | 8 | 29 |
| DNA binding | 4 | 0 | 1 | 1 | 2 | 5 | 6 | 19 |
| hydrolase activity | 5 | 3 | 2 | 2 | 5 | 5 | 0 | 22 |
| metal ion binding | 10 | 0 | 4 | 2 | 1 | 1 | 5 | 23 |
| molecular_function | 3 | 0 | 6 | 0 | 5 | 2 | 3 | 19 |
| nucleic acid binding | 4 | 1 | 5 | 2 | 0 | 1 | 6 | 19 |
| nucleotide binding | 16 | 2 | 5 | 4 | 6 | 10 | 9 | 52 |
| oxidoreductase activity | 9 | 0 | 1 | 3 | 0 | 3 | 2 | 18 |
| protein binding | 14 | 1 | 9 | 8 | 11 | 8 | 8 | 59 |
| protein kinase activity | 8 | 0 | 1 | 2 | 3 | 3 | 4 | 21 |
| protein serine/threonine kinase activity | 8 | 0 | 0 | 2 | 3 | 3 | 3 | 19 |
| RNA binding | 3 | 1 | 2 | 2 | 0 | 1 | 3 | 12 |
| transferase activity | 6 | 0 | 3 | 0 | 2 | 1 | 3 | 15 |
| transferase activity, transferring phosphorus-containing groups | 8 | 0 | 1 | 2 | 3 | 3 | 4 | 21 |
| zinc ion binding | 7 | 0 | 4 | 4 | 2 | 4 | 5 | 26 |
| Total | 129 | 12 | 52 | 43 | 56 | 64 | 80 | 436 |
