# Supplementary material for: Quantitative Trait Loci for Yield and Yield-Related Traits in Spring Barley Populations Derived from Crosses between European and Syrian Cultivars
Source: PLoS One. 2016 May 26;11(5):e0155938. doi: 10.1371/journal.pone.0155938 (PMC4881963; doi:10.1371/journal.pone.0155938)
Supplement: S4 Table — (DOCX) [file pone.0155938.s005.docx]

S4 Table. Results of stability analysis for RIL populations MCam and LCam. Marked are lines with 5880-2547 alleles inherited from Cam/B1/CI (early heading. allele "2") and 5260-462 alleles inherited from Maresi or 4026-655 alleles inherited form Lubuski (*denso* phenotype. allele "1"). In yellow - lines which are stable (GE interaction not significant) and do not have a significantly negative mean genotypic effect; in green - lines which are not stable (GE interaction significant) and have a negative and significant regression of genotypic effects in consecutive years on environmental effects (all tests at

P < 0.05)

| MCam line | Genotypic effect in 2011 | Genotypic effect in 2012 | Genotypic effect in 2013 | Mean genotypic effect | P-value for mean effect | P-value for GE interaction | Regression coefficient | P-value for regression | Genotype in 5880-2547 | Genotype in 5260-462 |
| --- | --- | --- | --- | --- | --- | --- | --- | --- | --- | --- |
| MCam001 | 20.23 | 13.00 | -18.07 | 5.05 | 0.709 | 0.758 | -0.036 | 0.658 | 1 | 1 |
| MCam002 | -2.27 | -77.00 | -1.40 | -26.95 | 0.394 | 0.284 | -0.095 | 0.569 | 1 | 1 |
| MCam004 | -24.77 | -20.30 | -36.40 | -27.17 | 0.030 | 0.955 | -0.003 | 0.944 | 1 | 1 |
| MCam005 | 23.56 | 111.30 | -51.40 | 27.83 | 0.614 | 0.012 | 0.059 | 0.866 | 1 | 1 |
| MCam006 | 7.73 | -82.00 | 28.60 | -15.29 | 0.696 | 0.100 | -0.100 | 0.676 | 1 | 1 |
| MCam007 | -22.27 | -153.70 | -34.73 | -70.28 | 0.235 | 0.030 | -0.177 | 0.508 | 1 | 1 |
| MCam008 | 11.06 | 141.30 | -1.40 | 50.27 | 0.386 | 0.016 | 0.158 | 0.611 | 1 | 1 |
| MCam009 | -9.77 | -170.30 | -161.40 | -113.84 | 0.160 | 0.004 | -0.313 | 0.073 |  | 1 |
| MCam010 | 19.40 | 28.00 | -84.73 | -12.51 | 0.763 | 0.072 | -0.062 | 0.816 | 1 | 1 |
| MCam014 | -10.60 | 169.70 | -43.07 | 38.60 | 0.619 | <0.001 | 0.208 | 0.652 | 1 | 1 |
| MCam017 | 21.06 | 181.30 | -174.73 | 9.16 | 0.937 | 0.000 | 0.067 | 0.931 | 1 | 2 |
| MCam021 | 19.40 | 76.30 | -36.40 | 19.83 | 0.604 | 0.120 | 0.033 | 0.892 | 1 | 2 |
| MCam024 | 0.23 | 94.70 | -78.07 | 5.60 | 0.921 | 0.007 | 0.066 | 0.861 | 1 | 1 |
| MCam026 | 33.56 | -13.70 | -133.07 | -37.73 | 0.526 | 0.007 | -0.178 | 0.594 | 1 | 1 |
| MCam027 | 4.40 | -32.00 | 25.93 | -0.62 | 0.974 | 0.563 | -0.031 | 0.804 | 1 | 1 |
| MCam028 | -20.60 | 101.30 | -31.40 | 16.49 | 0.736 | 0.027 | 0.148 | 0.609 | 1 | 2 |
| MCam035 | 5.23 | 201.30 | 30.27 | 78.94 | 0.329 | <0.001 | 0.269 | 0.487 | 1 | 2 |
| MCam039 | -1.44 | 116.30 | -21.40 | 31.16 | 0.544 | 0.025 | 0.137 | 0.647 | 1 | 1 |
| MCam041 | 36.90 | 89.70 | 191.93 | 106.16 | 0.145 | 0.016 | 0.177 | 0.554 | 1 | 1 |
| MCam042 | 4.40 | 249.70 | 1.93 | 85.38 | 0.408 | <0.001 | 0.312 | 0.568 | 1 |  |
| MCam043 | -12.27 | -257.00 | 21.60 | -82.51 | 0.447 | <0.001 | -0.290 | 0.632 | 2 | 1 |
| MCam044 | 1.06 | -137.00 | 113.93 | -7.40 | 0.928 | <0.001 | -0.097 | 0.859 | 2 | 2 |
| MCam045 | -14.77 | -157.00 | 91.93 | -26.62 | 0.747 | <0.001 | -0.107 | 0.843 | 1 | 1 |
| MCam046 | -27.27 | -113.70 | 31.93 | -36.40 | 0.480 | 0.028 | -0.068 | 0.828 | 2 | 2 |
| MCam048 | -14.77 | 53.00 | -8.07 | 10.05 | 0.687 | 0.393 | 0.092 | 0.505 | 1 | 1 |
| MCam049 | -1.44 | 18.00 | 88.60 | 35.05 | 0.329 | 0.223 | 0.089 | 0.640 | 1 | 1 |
| MCam050 | -18.94 | -128.70 | 71.93 | -25.17 | 0.707 | 0.001 | -0.077 | 0.860 | 1 | 1 |
| MCam052 | 7.73 | -42.00 | 55.27 | 6.94 | 0.828 | 0.205 | -0.030 | 0.888 | 1 | 1 |
| MCam053 | 4.40 | -38.70 | 68.60 | 11.38 | 0.750 | 0.142 | -0.009 | 0.968 | 2 | 1 |
| MCam056 | -27.27 | 26.30 | -51.40 | -17.40 | 0.528 | 0.347 | 0.051 | 0.759 | 1 | 1 |
| MCam057 | -13.10 | 1.30 | -78.07 | -29.95 | 0.345 | 0.302 | -0.028 | 0.881 | 1 | 1 |
| MCam058 | 38.56 | 14.70 | 95.27 | 49.49 | 0.174 | 0.317 | 0.010 | 0.958 | 1 | 1 |
| MCam059 | -16.44 | -92.00 | 95.27 | -4.40 | 0.943 | 0.003 | -0.018 | 0.966 | 1 | 1 |
| MCam060 | 5.23 | -28.70 | 105.27 | 27.27 | 0.568 | 0.039 | 0.027 | 0.928 | 1 | 1 |
| MCam061 | 44.40 | 106.30 | -8.07 | 47.60 | 0.287 | 0.112 | 0.042 | 0.866 | 1 | 1 |
| MCam063 | -25.60 | 33.00 | 21.93 | 9.83 | 0.638 | 0.524 | 0.108 | 0.009 | 1 | 1 |
| MCam065 | 6.06 | 53.00 | -30.40 | 9.60 | 0.729 | 0.311 | 0.034 | 0.851 | 1 | 1 |
| MCam066 | -41.44 | 174.70 | 66.93 | 66.72 | 0.397 | <0.001 | 0.354 | 0.227 | 1 | 1 |
| MCam067 | -18.10 | -92.00 | -54.73 | -54.95 | 0.123 | 0.401 | -0.121 | 0.232 | 2 | 1 |
| MCam068 | -20.60 | 3.00 | -98.07 | -38.51 | 0.334 | 0.154 | -0.025 | 0.914 | 2 | 2 |
| MCam069 | -1.44 | -15.30 | -101.40 | -39.40 | 0.335 | 0.141 | -0.089 | 0.690 | 2 | 2 |
| MCam070 | -13.94 | -52.00 | -44.73 | -36.95 | 0.086 | 0.762 | -0.070 | 0.011 | 2 | 2 |
| MCam071 | 1.06 | -167.00 | -143.73 | -103.17 | 0.189 | 0.004 | -0.318 | 0.023 | 2 | 1 |
| MCam072 | 6.06 | -127.00 | -111.07 | -77.28 | 0.207 | 0.029 | -0.254 | 0.035 | 2 | 2 |
| MCam073 | -35.60 | -100.30 | -64.73 | -66.84 | 0.071 | 0.493 | -0.104 | 0.264 | 2 | 2 |
| MCam074 | -9.77 | -128.70 | -12.07 | -50.17 | 0.330 | 0.046 | -0.154 | 0.551 | 2 | 1 |
| MCam075 | -1.44 | 1.30 | 55.27 | 18.38 | 0.424 | 0.504 | 0.044 | 0.744 | 2 | 1 |
| MCam076 | 12.73 | -355.30 | 101.93 | -80.17 | 0.625 | <0.001 | -0.409 | 0.679 | 1 | 1 |
| MCam077 | -9.77 | -93.70 | -46.40 | -49.95 | 0.176 | 0.306 | -0.133 | 0.275 | 2 | 2 |
| MCam078 | -4.77 | 213.00 | -38.07 | 56.72 | 0.546 | <0.001 | 0.255 | 0.640 | 1 | 1 |
| MCam079 | -7.27 | -212.00 | -58.07 | -92.51 | 0.272 | <0.001 | -0.298 | 0.409 | 2 | 1 |
| MCam080 | -7.27 | -75.30 | -34.73 | -39.06 | 0.187 | 0.454 | -0.107 | 0.298 | 2 | 1 |
| MCam081 | 24.40 | 88.00 | 65.27 | 59.16 | 0.087 | 0.497 | 0.111 | 0.124 | 1 | 1 |
| MCam082 | -2.27 | 293.00 | -18.07 | 90.83 | 0.464 | <0.001 | 0.367 | 0.590 | 1 | 1 |
| MCam084 | 4.40 | 48.00 | -8.07 | 14.71 | 0.478 | 0.559 | 0.047 | 0.696 | 1 | 2 |
| MCam085 | -14.77 | 99.70 | 1.93 | 29.05 | 0.500 | 0.079 | 0.158 | 0.477 | 1 | 2 |
| MCam086 | -29.77 | -33.70 | -131.40 | -64.95 | 0.190 | 0.109 | -0.077 | 0.750 | 1 | 1 |
| MCam087 | 21.06 | 58.00 | 61.93 | 46.94 | 0.070 | 0.709 | 0.077 | 0.160 | 2 | 1 |
| MCam088 | -51.44 | -215.30 | -114.73 | -127.17 | 0.117 | 0.011 | -0.255 | 0.312 | 2 | 2 |
| MCam089 | -27.27 | -172.00 | -105.73 | -101.73 | 0.135 | 0.030 | -0.241 | 0.198 | 2 | 2 |
| MCam090 | -5.60 | 13.00 | -108.07 | -33.62 | 0.466 | 0.059 | -0.048 | 0.864 | 1 | 1 |
| MCam091 | 8.56 | 41.30 | -44.73 | 1.72 | 0.952 | 0.283 | 0.004 | 0.982 | 1 | 1 |
| MCam092 | 36.90 | 199.70 | -28.07 | 69.49 | 0.413 | <0.001 | 0.162 | 0.740 | 1 | 2 |
| MCam093 | -6.44 | -40.30 | 78.60 | 10.60 | 0.793 | 0.081 | 0.017 | 0.950 | 1 | 1 |
| MCam094 | 11.06 | -25.30 | -28.07 | -14.06 | 0.383 | 0.724 | -0.075 | 0.144 | 1 | 1 |
| MCam097 | 11.90 | -74.30 | 145.27 | 27.60 | 0.708 | <0.001 | -0.016 | 0.974 | 1 | 1 |
| MCam100 | -8.10 | 121.30 | -4.73 | 36.16 | 0.485 | 0.027 | 0.168 | 0.547 | 1 | 2 |
| MCam101 | -19.77 | 11.30 | 28.60 | 6.71 | 0.682 | 0.668 | 0.074 | 0.334 | 1 | 1 |
| MCam102 | -24.77 | 194.70 | -148.07 | 7.27 | 0.949 | <0.001 | 0.194 | 0.793 | 1 | 2 |
| MCam103 | 14.40 | -12.00 | -31.40 | -9.73 | 0.538 | 0.704 | -0.066 | 0.383 | 1 | 1 |
| MCam104 | -16.44 | -53.70 | 105.27 | 11.71 | 0.830 | 0.010 | 0.038 | 0.915 | 1 | 1 |
| MCam105 | 0.23 | -75.30 | 45.27 | -9.95 | 0.804 | 0.084 | -0.065 | 0.803 | 1 | 1 |
| MCam106 | -3.94 | -48.70 | -64.73 | -39.06 | 0.166 | 0.512 | -0.101 | 0.268 | 1 | 2 |
| MCam107 | 15.23 | 71.30 | -47.73 | 12.94 | 0.743 | 0.093 | 0.027 | 0.916 | 1 | 1 |
| MCam108 | 3.56 | -102.00 | 98.60 | 0.05 | 1.000 | 0.001 | -0.068 | 0.876 | 1 | 1 |
| MCam109 | 16.90 | 174.70 | 131.93 | 107.83 | 0.149 | 0.012 | 0.284 | 0.064 | 1 | 1 |
| MCam110 | 8.56 | 223.00 | 35.27 | 88.94 | 0.318 | <0.001 | 0.294 | 0.489 | 1 | 1 |
| MCam111 | 20.23 | -58.70 | 128.60 | 30.05 | 0.636 | 0.003 | -0.024 | 0.953 | 1 | 1 |
| MCam112 | -27.27 | -8.70 | -121.40 | -52.40 | 0.272 | 0.087 | -0.043 | 0.869 | 1 | 1 |
| MCam113 | 1.90 | -117.00 | 15.27 | -33.28 | 0.512 | 0.029 | -0.143 | 0.620 | 1 | 2 |
| MCam114 | 7.73 | -153.70 | 45.27 | -33.51 | 0.638 | <0.001 | -0.180 | 0.675 | 1 | 2 |
| MCam115 | 22.73 | 68.00 | -28.07 | 20.94 | 0.529 | 0.213 | 0.022 | 0.918 | 1 | 1 |
| MCam118 | 24.40 | 133.00 | 285.27 | 147.60 | 0.190 | <0.001 | 0.323 | 0.500 | 1 | 1 |
| MCam119 | 39.40 | -82.00 | 48.60 | 1.94 | 0.967 | 0.029 | -0.149 | 0.603 | 1 | 2 |
| MCam120 | 6.90 | -52.00 | -24.73 | -23.29 | 0.305 | 0.559 | -0.098 | 0.201 | 1 | 2 |
| MCam121 | -21.44 | 4.70 | 60.27 | 14.49 | 0.609 | 0.312 | 0.091 | 0.569 | 1 | 1 |
| MCam122 | 35.23 | 6.30 | 85.27 | 42.27 | 0.208 | 0.344 | -0.002 | 0.993 | 1 | 1 |
| MCam123 | -8.10 | 128.00 | 66.93 | 62.38 | 0.253 | 0.046 | 0.227 | 0.192 | 1 | 1 |
| MCam124 | 25.23 | 104.70 | -111.40 | 6.16 | 0.931 | <0.001 | 0.005 | 0.992 | 1 | 2 |
| MCam125 | -10.60 | 193.00 | 28.60 | 70.27 | 0.377 | <0.001 | 0.289 | 0.445 | 1 | 2 |
| MCam126 | -32.27 | -153.70 | -36.40 | -74.06 | 0.204 | 0.042 | -0.159 | 0.542 | 1 | 1 |
| MCam128 | 1.06 | -48.70 | 96.93 | 16.38 | 0.738 | 0.026 | 0.004 | 0.989 | 1 | 1 |
| MCam129 | 46.06 | 216.30 | 215.27 | 159.27 | 0.106 | 0.002 | 0.338 | 0.101 | 1 | 1 |
| MCam130 | 2.73 | -272.00 | -141.40 | -136.95 | 0.226 | <0.001 | -0.454 | 0.211 | 1 | 1 |
| LCam line | Genotypic effect in 2011 | Genotypic effect in 2012 | Genotypic effect in 2013 | Mean genotypic effect | P-value for mean effect | P-value for GE interaction | Regression coefficient | P-value for regression | Genotype in 5880-2547 | Genotype in 4026-655 |
| LCam002 | -25.06 | -47.90 | 118.30 | 15.07 | 0.799 | <0.001 | 0.061 | 0.882 | 1 | 1 |
| LCam004 | -28.39 | 117.10 | -41.70 | 15.63 | 0.788 | <0.001 | 0.199 | 0.579 | 1 | 1 |
| LCam005 | -21.73 | 95.50 | 18.30 | 30.63 | 0.468 | 0.034 | 0.194 | 0.313 | 1 | 1 |
| LCam006 | -20.06 | 75.50 | 98.30 | 51.18 | 0.294 | 0.023 | 0.214 | 0.251 | 1 | 1 |
| LCam007 | 19.11 | 97.10 | -145.10 | -9.71 | 0.904 | <0.001 | 0.005 | 0.993 | 1 | 1 |
| LCam008 | 16.61 | 73.80 | 71.60 | 53.96 | 0.103 | 0.364 | 0.118 | 0.114 | 1 | 1 |
| LCam009 | 6.61 | -41.20 | -111.70 | -48.82 | 0.291 | 0.034 | -0.145 | 0.539 | 1 | 1 |
| LCam010 | 5.77 | 128.80 | -31.70 | 34.18 | 0.554 | 0.001 | 0.152 | 0.674 | 1 | 1 |
| LCam011 | 7.44 | -187.90 | -61.70 | -80.82 | 0.293 | <0.001 | -0.323 | 0.305 | 1 | 1 |
| LCam012 | 24.94 | 190.50 | -35.10 | 60.07 | 0.467 | <0.001 | 0.198 | 0.696 | 1 | 1 |
| LCam013 | 20.77 | 205.50 | 151.60 | 125.96 | 0.148 | <0.001 | 0.349 | 0.048 | 1 | 1 |
| LCam014 | 5.77 | 37.10 | 54.90 | 32.63 | 0.151 | 0.553 | 0.077 | 0.368 | 1 | 1 |
| LCam015 | 27.44 | 50.50 | 34.90 | 37.63 | 0.031 | 0.876 | 0.038 | 0.326 | 1 | 1 |
| LCam016 | -28.39 | -21.20 | 201.60 | 50.74 | 0.571 | <0.001 | 0.160 | 0.785 | 1 | 1 |
| LCam017 | 25.77 | -79.50 | -108.40 | -54.15 | 0.315 | 0.009 | -0.237 | 0.267 | 1 | 1 |
| LCam018 | 33.27 | 50.50 | -81.70 | 0.63 | 0.989 | 0.007 | -0.050 | 0.879 | 1 | 2 |
| LCam020 | 4.94 | 117.10 | -31.70 | 30.07 | 0.571 | 0.003 | 0.137 | 0.683 | 1 | 1 |
| LCam021 | -5.06 | 172.10 | 154.90 | 107.29 | 0.198 | <0.001 | 0.358 | 0.079 | 1 | 1 |
| LCam023 | -1.73 | 78.80 | -11.70 | 21.74 | 0.528 | 0.095 | 0.109 | 0.595 | 1 | 1 |
| LCam024 | 4.11 | 80.50 | 218.30 | 100.96 | 0.248 | <0.001 | 0.248 | 0.573 | 1 | 1 |
| LCam025 | -10.89 | 42.10 | 324.90 | 118.74 | 0.373 | <0.001 | 0.294 | 0.708 | 1 | 1 |
| LCam026 | 12.44 | 290.50 | -45.10 | 85.96 | 0.494 | <0.001 | 0.360 | 0.634 | 1 | 2 |
| LCam027 | 12.44 | -152.90 | 98.30 | -14.15 | 0.865 | <0.001 | -0.180 | 0.751 | 2 |  |
| LCam029 | 44.11 | 30.50 | 354.90 | 143.07 | 0.31 | <0.001 | 0.184 | 0.825 | 1 | 1 |
| LCam030 | -14.23 | -92.90 | 144.90 | 12.63 | 0.873 | <0.001 | -0.009 | 0.988 | 1 | 1 |
| LCam031 | 15.77 | -32.90 | -188.40 | -68.48 | 0.382 | <0.001 | -0.203 | 0.655 | 1 | 1 |
| LCam032 | -7.56 | -19.50 | -108.40 | -45.15 | 0.292 | 0.055 | -0.083 | 0.732 | 1 | 1 |
| LCam033 | -2.56 | 90.50 | -5.10 | 27.63 | 0.472 | 0.059 | 0.131 | 0.546 | 1 | 1 |
| LCam035 | 11.61 | 62.10 | 61.60 | 45.07 | 0.115 | 0.444 | 0.105 | 0.129 | 1 | 1 |
| LCam036 | -2.56 | -31.20 | -51.70 | -28.60 | 0.181 | 0.561 | -0.072 | 0.410 | 1 | 1 |
| LCam037 | -52.14 | -31.20 | 18.30 | -18.64 | 0.425 | 0.364 | 0.057 | 0.686 | 2 | 2 |
| LCam038 | 28.27 | -21.20 | -151.70 | -48.15 | 0.465 | <0.001 | -0.188 | 0.630 | 2 | 1 |
| LCam039 | -12.56 | 248.80 | 111.60 | 115.85 | 0.265 | <0.001 | 0.455 | 0.216 | 1 | 1 |
| LCam040 | 1.61 | 12.10 | 171.60 | 61.74 | 0.378 | <0.001 | 0.126 | 0.766 | 1 | 1 |
| LCam041 | 29.94 | 292.10 | 98.30 | 140.07 | 0.217 | <0.001 | 0.419 | 0.370 | 1 | 1 |
| LCam042 | 6.61 | 133.80 | 208.30 | 116.18 | 0.187 | <0.001 | 0.313 | 0.373 | 1 | 1 |
| LCam043 | -3.39 | 8.80 | -68.40 | -21.04 | 0.472 | 0.193 | -0.025 | 0.897 |  | 1 |
| LCam045 | -37.56 | -142.90 | -25.10 | -68.60 | 0.207 | 0.019 | -0.142 | 0.594 | 1 | 1 |
| LCam046 | 8.27 | -14.50 | 78.30 | 23.96 | 0.481 | 0.107 | 0.013 | 0.953 | 1 | 1 |
| LCam047 | -30.06 | 160.50 | -111.70 | 6.18 | 0.946 | <0.001 | 0.219 | 0.720 | 1 | 1 |
| LCam048 | -14.23 | 20.50 | -18.40 | -4.15 | 0.769 | 0.644 | 0.047 | 0.589 | 1 | 1 |
| LCam049 | -17.56 | -159.50 | -145.10 | -107.48 | 0.14 | 0.003 | -0.285 | 0.076 | 1 | 1 |
| LCam050 | -8.39 | -214.50 | -181.70 | -134.93 | 0.169 | <0.001 | -0.407 | 0.041 | 2 | 1 |
| LCam051 | -0.06 | 7.10 | -108.40 | -33.82 | 0.461 | 0.018 | -0.060 | 0.838 | 1 | 1 |
| LCam052 | 22.44 | 62.10 | -148.40 | -21.26 | 0.773 | <0.001 | -0.055 | 0.915 | 1 | 1 |
| LCam053 | 3.27 | -19.50 | -98.40 | -38.26 | 0.34 | 0.066 | -0.099 | 0.665 |  | 1 |
| LCam054 | 17.44 | 147.10 | 64.90 | 76.40 | 0.182 | 0.016 | 0.217 | 0.295 | 1 | 2 |
| LCam055 | -9.23 | -29.50 | -8.40 | -15.71 | 0.151 | 0.871 | -0.029 | 0.552 | 1 | 1 |
| LCam056 | 4.11 | 35.50 | 8.30 | 15.96 | 0.246 | 0.757 | 0.047 | 0.454 | 1 | 1 |
| LCam057 | 8.27 | -87.90 | -25.10 | -34.93 | 0.34 | 0.103 | -0.159 | 0.310 | 2 | 1 |
| LCam059 | 10.77 | 158.80 | 1.60 | 56.96 | 0.38 | <0.001 | 0.206 | 0.563 | 1 | 1 |
| LCam060 | -16.73 | -164.50 | -128.40 | -103.26 | 0.146 | 0.004 | -0.284 | 0.015 |  | 1 |
| LCam061 | -16.73 | -117.90 | -51.70 | -62.15 | 0.171 | 0.081 | -0.167 | 0.311 | 2 | 1 |
| LCam062 | 2.44 | -132.90 | -178.40 | -103.04 | 0.198 | <0.001 | -0.310 | 0.291 | 2 | 1 |
| LCam063 | -15.89 | -34.50 | 154.90 | 34.85 | 0.622 | <0.001 | 0.085 | 0.859 | 1 | 1 |
| LCam064 | 0.77 | -236.20 | 1.60 | -78.04 | 0.428 | <0.001 | -0.337 | 0.534 | 2 | 1 |
| LCam065 | -11.73 | -157.90 | -108.40 | -92.71 | 0.163 | 0.005 | -0.271 | 0.081 | 2 | 2 |
| LCam067 | -20.89 | -131.20 | 54.90 | -32.37 | 0.61 | <0.001 | -0.108 | 0.797 | 1 | 1 |
| LCam068 | 5.77 | -139.50 | -235.10 | -122.93 | 0.221 | <0.001 | -0.364 | 0.393 | 1 | 1 |
| LCam069 | -3.39 | 37.10 | -135.10 | -33.82 | 0.582 | <0.001 | -0.028 | 0.947 | 1 | 1 |
| LCam070 | -5.89 | -332.90 | -45.10 | -127.93 | 0.34 | <0.001 | -0.493 | 0.461 | 2 | 1 |
| LCam071 | -6.73 | -157.90 | -135.10 | -99.82 | 0.168 | 0.002 | -0.300 | 0.046 | 2 | 1 |
| LCam072 | 29.11 | -124.50 | -71.70 | -55.82 | 0.34 | 0.003 | -0.284 | 0.085 | 2 | 1 |
| LCam073 | -30.89 | -252.90 | 94.90 | -63.04 | 0.598 | 0.000 | -0.234 | 0.765 | 2 | 1 |
| LCam074 | 16.61 | 137.10 | 128.30 | 93.96 | 0.137 | 0.013 | 0.245 | 0.093 | 1 | 1 |
| LCam075 | 9.94 | -224.50 | -148.40 | -121.04 | 0.222 | <0.001 | -0.438 | 0.071 | 2 |  |
| LCam076 | -15.89 | -381.20 | 174.90 | -74.15 | 0.694 | <0.001 | -0.397 | 0.751 | 2 | 1 |
| LCam077 | -16.73 | -122.90 | -98.40 | -79.37 | 0.132 | 0.053 | -0.205 | 0.006 | 2 | 1 |
| LCam078 | -3.39 | 93.80 | 131.60 | 73.96 | 0.207 | 0.010 | 0.227 | 0.310 | 1 | 1 |
| LCam079 | -12.56 | 102.10 | 38.30 | 42.52 | 0.329 | 0.042 | 0.198 | 0.239 | 1 | 1 |
| LCam080 | -5.06 | -49.50 | 18.30 | -12.15 | 0.603 | 0.321 | -0.048 | 0.753 | 1 | 1 |
| LCam081 | -21.73 | 27.10 | 114.90 | 40.07 | 0.422 | 0.010 | 0.159 | 0.572 | 1 | 1 |
| LCam082 | 12.44 | 237.10 | -65.10 | 61.40 | 0.568 | <0.001 | 0.271 | 0.690 | 1 | 1 |
| LCam083 | 41.61 | 153.80 | 138.30 | 111.18 | 0.087 | 0.029 | 0.223 | 0.054 | 1 | 1 |
| LCam085 | -29.23 | -262.90 | -75.10 | -122.37 | 0.229 | <0.001 | -0.364 | 0.413 | 1 | 1 |
| LCam087 | 11.61 | -64.50 | 28.30 | -8.15 | 0.802 | 0.096 | -0.098 | 0.638 | 2 | 1 |
| LCam088 | -35.06 | -189.50 | -165.10 | -129.93 | 0.113 | 0.001 | -0.305 | 0.041 | 1 | 1 |
| LCam089 | -5.89 | -37.90 | 181.60 | 45.96 | 0.571 | <0.001 | 0.076 | 0.888 | 2 | 1 |
| LCam090 | 18.27 | -36.20 | -81.70 | -33.26 | 0.368 | 0.092 | -0.143 | 0.436 | 1 | 1 |
| LCam091 | 3.27 | 115.50 | -51.70 | 22.40 | 0.693 | 0.001 | 0.124 | 0.742 | 1 | 1 |
| LCam092 | -6.73 | 142.10 | -81.70 | 17.85 | 0.812 | <0.001 | 0.164 | 0.745 | 1 | 1 |
| LCam093 | 12.44 | 195.50 | 201.60 | 136.40 | 0.159 | <0.001 | 0.385 | 0.153 | 1 | 2 |
| LCam094 | 4.94 | -84.50 | 11.60 | -22.71 | 0.54 | 0.064 | -0.123 | 0.572 | 1 | 1 |
| LCam095 | -22.56 | 58.80 | -75.10 | -13.04 | 0.77 | 0.013 | 0.083 | 0.784 | 1 | 1 |
| LCam096 | 9.11 | -54.50 | -238.40 | -94.60 | 0.331 | <0.001 | -0.252 | 0.643 | 1 | 1 |
| LCam097 | 29.11 | 53.80 | 24.90 | 35.96 | 0.057 | 0.792 | 0.032 | 0.618 | 1 | 1 |
| LCam099 | -10.06 | 113.80 | -88.40 | 5.07 | 0.939 | 0.000 | 0.126 | 0.782 | 2 | 1 |
| LCam100 | 19.94 | 77.10 | 41.60 | 46.18 | 0.11 | 0.448 | 0.096 | 0.286 | 1 | 1 |
